# Mouse BAZ1A (ACF1) Is Dispensable for Double-Strand Break Repair but Is Essential for Averting Improper Gene Expression during Spermatogenesis

**DOI:** 10.1371/journal.pgen.1003945

**Published:** 2013-11-07

**Authors:** James A. Dowdle, Monika Mehta, Elizabeth M. Kass, Bao Q. Vuong, Akiko Inagaki, Dieter Egli, Maria Jasin, Scott Keeney

**Affiliations:** 1 Louis V. Gerstner Jr. Graduate School of Biomedical Sciences, New York, New York, United States of America; 2 Molecular Biology Program, Memorial Sloan-Kettering Cancer Center, New York, New York, United States of America; 3 Howard Hughes Medical Institute, Memorial Sloan-Kettering Cancer Center, New York, New York, United States of America; 4 Developmental Biology Program, Memorial Sloan-Kettering Cancer Center, New York, New York, United States of America; 5 Immunology Program, Memorial Sloan-Kettering Cancer Center, New York, New York, United States of America; 6 The New York Stem Cell Foundation, New York, New York, United States of America; Cornell University, United States of America

## Abstract

ATP-dependent chromatin remodelers control DNA access for transcription, recombination, and other processes. Acf1 (also known as BAZ1A in mammals) is a defining subunit of the conserved ISWI-family chromatin remodelers ACF and CHRAC, first purified over 15 years ago from *Drosophila melanogaster* embryos. Much is known about biochemical properties of ACF and CHRAC, which move nucleosomes *in vitro* and *in vivo* to establish ordered chromatin arrays. Genetic studies in yeast, flies and cultured human cells clearly implicate these complexes in transcriptional repression via control of chromatin structures. RNAi experiments in transformed mammalian cells in culture also implicate ACF and CHRAC in DNA damage checkpoints and double-strand break repair. However, their essential *in vivo* roles in mammals are unknown. Here, we show that *Baz1a*-knockout mice are viable and able to repair developmentally programmed DNA double-strand breaks in the immune system and germ line, I-SceI endonuclease-induced breaks in primary fibroblasts via homologous recombination, and DNA damage from mitomycin C exposure *in vivo*. However, *Baz1a* deficiency causes male-specific sterility in accord with its high expression in male germ cells, where it displays dynamic, stage-specific patterns of chromosomal localization. Sterility is caused by pronounced defects in sperm development, most likely a consequence of massively perturbed gene expression in spermatocytes and round spermatids in the absence of BAZ1A: the normal spermiogenic transcription program is largely intact but more than 900 other genes are mis-regulated, primarily reflecting inappropriate up-regulation. We propose that large-scale changes in chromatin composition that occur during spermatogenesis create a window of vulnerability to promiscuous transcription changes, with an essential function of ACF and/or CHRAC chromatin remodeling activities being to safeguard against these alterations.

## Author Summary

The eukaryotic genome is packaged into a periodic nucleoprotein complex known as chromatin. Wrapping of DNA around nucleosomes, the basic repeat unit of chromatin, enables packing of long stretches of DNA into a compact nucleus but also impedes access by protein factors involved in essential cellular processes such as transcription, replication, recombination and repair. Chromatin remodeling factors are multi-protein complexes that utilize the energy released during ATP-hydrolysis to assemble, reposition, restructure and disassemble nucleosomes. These complexes disrupt histone-DNA contacts to ‘remodel’ the chromatin and grant access to the genome. Alternatively, access can also be denied to repress transcription, for example. Spermatogenesis, the developmental program that produces sperm, comprises a dramatic chromatin makeover and the induction of a transcriptional program that engages nearly one-third of the genome. Here we provide evidence suggesting that these large-scale alterations leave the genomic material vulnerable to spurious transcriptional changes which are normally repressed by ACF1 (BAZ1A in mammals), the defining member of the well-studied ACF/CHRAC chromatin remodeling complex. These findings indicate that *Baz1a* plays a previously unrealized role in male fertility and may represent a novel target for male contraceptive development.

## Introduction

The nucleosome, a complex of eight histone proteins wrapped by 146 bp of DNA, is a fundamental packaging unit for nuclear DNA, controlling access by proteins involved in transcription, replication, recombination and repair. Granting or blocking DNA access can be effected through changes in histone-DNA contacts by action of chromatin remodelers, ATP-dependent multi-protein complexes that assemble, reposition, restructure and/or disassemble nucleosomes [1], [2]. Each remodeler comprises an ATPase of the Swi2/Snf2 family of helicases/translocases and one or more accessory factors that confer biological specificity by modulating the ATPase's activity and/or targeting to particular genomic locations.

ACF and CHRAC are particularly well-studied examples of the widely conserved imitation switch (ISWI) family of remodelers, first purified from *Drosophila* [3], [4]. *Drosophila* ACF (ATP-dependent chromatin assembly and remodeling factor) is a two-subunit complex containing the ATPase ISWI bound to Acf1 [5]. Its human counterpart contains the ISWI homolog SNF2H (SMARCA5) and the Acf1 homolog BAZ1A (also known as ACF1) [6], [7]. CHRAC (chromatin accessibility complex) is a larger version of ACF which, in addition to Acf1 and ISWI, contains two small histone-fold proteins: CHRAC14 and -16 in *Drosophila* [8], [9], CHRAC15 and -17 in human [10] (Figure S1A). BAZ1A is so named because it shares domain architecture with other BAZ family members: a bromodomain adjacent to a zinc finger, typically a plant homeo domain (PHD) [11] (Figure S1B). *Drosophila* and mammals have multiple Acf1 homologs, each of which associates with an ATPase with or without additional proteins to form a large family of distinct ISWI chromatin remodeling complexes (Figure S1A). Of the seven Acf1 homologs in mammals, BAZ1A is the one most similar to *Drosophila* Acf1 (Figure S1C), so it is a defining subunit of mammalian ACF and CHRAC complexes.

A great deal is known about the enzymatic activities of ACF and CHRAC because the human and *Drosophila* proteins, and the equivalent ISW2 complex of budding yeast, have been paradigms for biochemical studies of ISWI complexes [reviewed in 12]–[14]. *In vitro*, they catalyze movements of nucleosomes to form regularly spaced nucleosome arrays, consistent with a principal function in assembly and maintenance of properly ordered chromatin structures [3], [4]. Much is also known about the biochemical functions of the non-ATPase subunits. Acf1/BAZ1A (and/or their yeast homolog Itc1) directly contacts nucleosome-adjacent linker DNA, increases the affinity and processivity of the ATPase on nucleosomal substrates, and alters basal ISWI/SNF2H nucleosome spacing and positioning activities *in vitro* [5], [8], [15]–[18]. The CHRAC proteins heterodimerize and directly bind both DNA and the N-terminus of Acf1/BAZ1A to further enhance ACF performance [15], [19]–[21]. Bulk biochemical, single-molecule, and structural studies have elucidated interactions of ACF and CHRAC with ATP, DNA, and nucleosomes, leading to detailed mechanistic models for their function in chromatin assembly and nucleosome movement [reviewed in 2], [12]–[14].

In contrast, comparatively little is known about these complexes' physiological roles in metazoans [22]. *Iswi* and *Snf2h* mutations cause embryonic lethality in flies and mice, respectively [23], [24], but these ATPases are the catalytic cores of multiple chromatin remodeling complexes with distinct biochemical properties and biological specificities [2], [22] (Figure S1A). Thus far, *Drosophila* genetic studies provide the only *in vivo* data specific to ACF/CHRAC. Null *acf1* mutations cause substantial lethality at larval stage L3, although about 25% of *acf1* flies survive to adulthood and are fertile, suggesting the existence of compensatory factors [25]. Mutants display less ordered nucleosome arrays, reduced position effect variegation, defects in establishment of pericentric heterochromatin and Polycomb-dependent repressive chromatin, and derepression of Wingless-regulated genes [25]–[27]. These properties suggest that ACF and/or CHRAC promote proper chromatin assembly *in vivo*, also supported by studies of homologous protein complexes in budding yeast [28]. RNAi depletion studies in cultured, tumor-derived human cells indicate that *Baz1a* facilitates replication through heterochromatic DNA [29], contributes to repression of vitamin D3 receptor-regulated genes [30], and promotes DNA double-strand break (DSB) repair [31] and the G2/M DNA damage checkpoint [32]. These findings may also reflect chromatin assembly functions of ACF/CHRAC, but whether these cellular roles are essential *in vivo* has not been addressed.

Of necessity, chromatin assembly occurs on a large scale during DNA replication. It has been suggested that ACF and/or CHRAC are especially critical at this time, in part because expression of *Drosophila* Acf1 is highest and its function most crucial during the rapid divisions of early embryonic development [25], [26]. However, chromatin assembly also occurs in non-replicative contexts. For example, developmentally programmed, post-replicative chromatin changes are a hallmark of mammalian spermatogenesis, including altered histone modifications, substitution of canonical histones with histone variants (many of which are testis-specific), and, late in spermiogenesis, replacement of most histones with transition proteins and then protamines [33]. Some of these alterations occur genome-wide, while some are targeted to specific regions such as sex chromosomes or transposable elements. Contemporaneously, gene expression is substantially rewired in successive waves to execute each spermatogenic stage [34]. Many factors that implement these normal programs of chromatin alteration and gene expression changes have been discovered, e.g., transcription factors CREM and MYBL1 and the histone H4 hyperacetylation reader BRDT [35]–[37]. However, given the profound influence of chromatin structure on gene expression, it is an open question how the extensive chromatin remodeling during spermatogenesis affects transcription.

Here, we examine the physiological functions of mammalian ACF and CHRAC by generating mice with a targeted *Baz1a* mutation. Surprisingly, *Baz1a* is dispensable for embryonic development, postnatal viability, and development of cells that experience programmed DSBs. Instead, BAZ1A is essential for proper spermiogenesis and thus male fertility, with BAZ1A required (directly or indirectly) to prevent inappropriate gene expression during periods of large-scale chromatin restructuring in spermatogenesis.

## Results

### BAZ1A protein expression and localization

**Figure 1:**
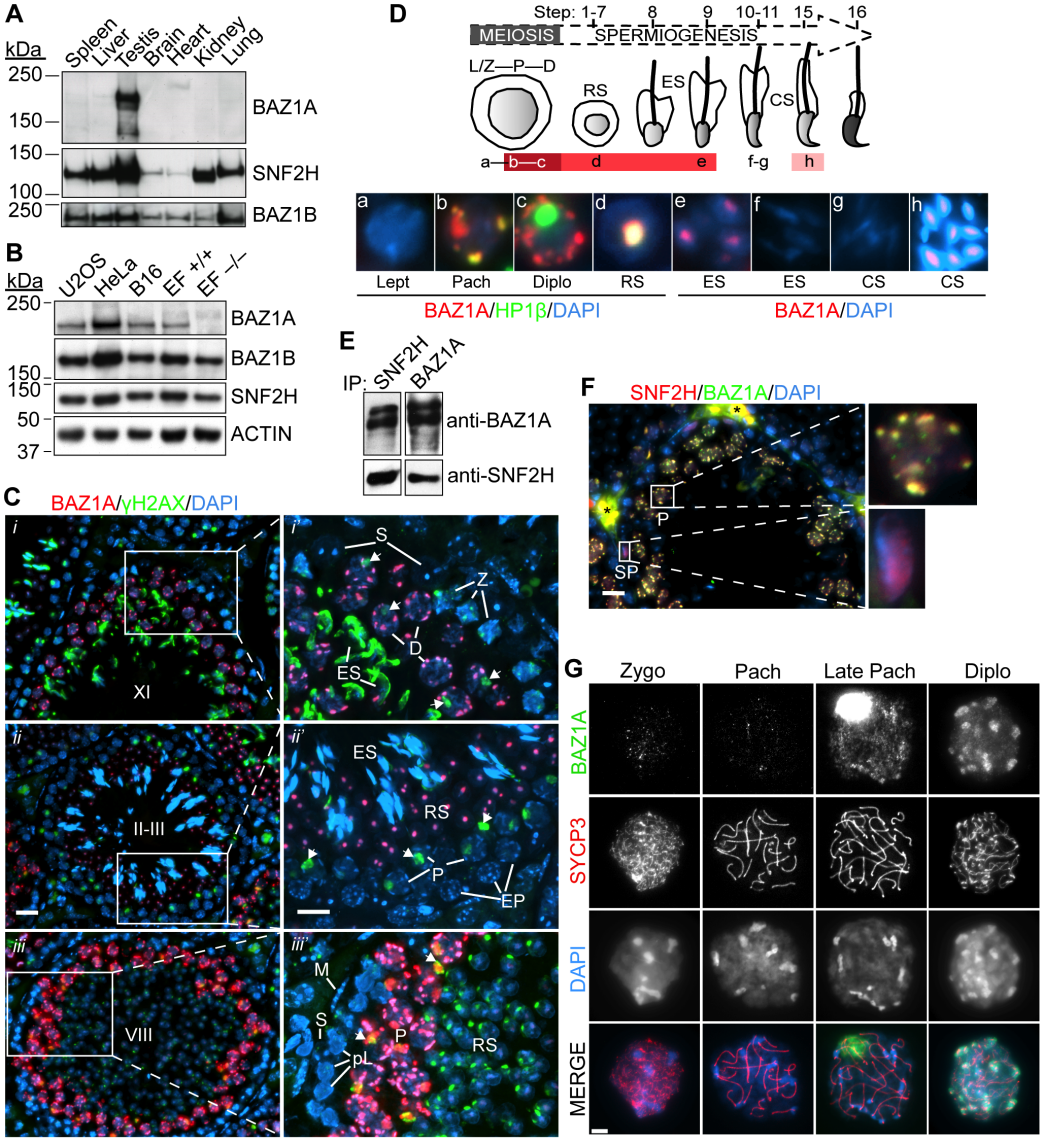
Expression and localization of BAZ1A in male germ cells. (A, B) Equal amounts of low-speed centrifuged, nuclear-enriched extracts from a panel of mouse tissues (8.5 µg protein) (A) or cultured human (U2OS and HeLa) and mouse (B16 and ear fibroblasts (EF)) cells (25 µg) (B) were immunoblotted with anti-BAZ1A, SNF2H and BAZ1B antibodies. Approximate molecular masses: BAZ1A = 178 kDa, SNF2H = 122 kDa, BAZ1B = 171 kDa and ACTIN = 42 kDa. +/+, wild-type; −/−, *Baz1a^−/−^*. (C) Immunofluorescence of testis sections with BAZ1A and γH2AX antibodies. (i–iii) Testis sections at various stages (indicated by uppercase Roman numerals). Bar = 20 µm. (i′–iii′) Magnification of areas indicated in left panels. Bar = 10 µm. Arrows point to sex bodies. Note that anti-γH2AX staining along the heads of elongating spermatids at stage X-XI and the focal signal in round spermatids at stage VIII is non-specific due to cross-reactivity with the developing acrosome. ES, elongating spermatid; RS, round spermatid; Z, zygotene spermatocyte; EP, early pachytene spermatocyte; S, Sertoli cell; P, pachytene spermatocyte; L, leptotene spermatocyte; pL, preleptotene spermatocyte; M, peritubular myoid cell; D, diplotene spermatocyte. (D) Schematic summarizing BAZ1A expression during spermatogenesis, with darker shades of red indicating intensity of BAZ1A staining. Micrographs are from insets in Figure S2B; panels a–d are co-stained with HP1β, a marker of heterochromatin (see Figure S2B and text for further explanation); panels e–h display higher exposures of the BAZ1A staining. RS, round spermatid; ES, elongating spermatid; CS, condensing spermatid. Abbreviations of meiotic stages as in (C). See text for full description of staining patterns. (E) Coimmunoprecipitation (IP) of SNF2H and BAZ1A from wild-type whole testis lysates. (F) Immunofluorescence of testis sections with BAZ1A and SNF2H antibodies. Magnifications of the indicated cells are shown. SP, spermatogonia; *autofluorescent red blood cells; bar = 20 µm. (G) BAZ1A immunofluorescence on squashed spermatocyte nuclei at different stages of meiotic prophase I as indicated by staining for the axial element component SYCP3. Bar = 5 µm.

BAZ1A-containing complexes (ACF and CHRAC) have been purified from cultured human cells, but their distribution *in vivo* had not been addressed. Human *BAZ1A* transcript levels are high in the testis compared to other tissues [11]. The same is true at the protein level in mice: BAZ1A was readily detected by immunoblotting of testis extracts but was below detection in other adult tissues examined (Figure 1A). Conversely, its paralog BAZ1B was readily detected in all tissues (Figure 1A). SNF2H was highly expressed in testis and showed strong expression in other tissues (Figure 1A). BAZ1A is likely produced in tissues other than testis, however, as it could be detected using the same western blot conditions in more concentrated extracts of cultured human and mouse cells (Figure 1B) and by RT-PCR from a panel of mouse tissues (data not shown).

To more precisely define BAZ1A expression and localization, we immunostained testis sections. As detailed below, expression was first detected in germ cells late in meiotic prophase, and continued through early steps of postmeiotic spermatid differentiation. BAZ1A abundance and sub-nuclear localization patterns were highly dynamic across this developmental timeline.

Spermatogenesis begins from spermatogonia, stem cells residing near the basal lamina of seminiferous tubules. Differentiation to primary spermatocytes is accompanied by migration toward the tubule lumen and onset of meiosis, in which one round of DNA replication precedes two rounds of chromosome segregation to generate haploid cells. Postmeiotic differentiation (spermiogenesis) converts these meiotic products into mature sperm. The progression of spermatogenesis in semi-synchronous waves allows classification of histological sections of the seminiferous epithelium into 12 stages based on their cellular content, which in turn allows the developmental timing of germ cells to be inferred (Figure S2A).

BAZ1A was not detected in leptotene/zygotene spermatocytes (e.g., in stage XI tubules; Figure 1C *i′*) or early pachytene spermatocytes (e.g., in stage II–III tubules; Figure 1C *ii′*), but was detectable in mid-pachytene spermatocytes starting in stage V (data not shown), becoming strongly stained in later-stage pachytene spermatocytes in stage VIII, IX, and X tubules where it was particularly enriched in DAPI-dense pericentric heterochromatin (Figure 1C *iii*′, Figure S2B *i*, S2B *ii*, and data not shown) and the sex body (see below). It was also enriched in pericentric heterochromatin but not sex bodies of diplotene spermatocytes in stage XI tubules (Figure 1C *i′*), in the chromocenters of round spermatids at stage II–III (Figure 1C *ii′*), and weakly in the further developed spermatids at stages VIII and IX, immediately prior to and just beginning to elongate, respectively (Figure 1C *iii′*, Figure S2B *i′*, and data not shown). BAZ1A partially overlapped with HP1β, confirming enrichment in heterochromatin (Figure 1D and Figure S2B), consistent with localization in cultured cells [29], [38]. BAZ1A was not detected in somatic Sertoli and peritubular myoid cells present in all tubule stages (Figure 1C *i′–iii′*). Figure 1D summarizes BAZ1A expression during spermatogenesis: it was first detected in mid-pachytene spermatocytes and continued through step 9 spermatids; it was not detectable in elongating step 10–11 spermatids (Figure S2B *ii′* and *iii′*, insets), but was seen again later, in step 15 spermatids (Figure S2B *iv′*, inset). By contrast, BAZ1A staining was only weakly detectable in pachytene and diplotene/dictyate oocytes in sections of embryonic or early postnatal ovaries (Figure S2C). Thus, high-level BAZ1A expression in germ cells is specific for males.

BAZ1A and SNF2H showed reciprocal coimmunoprecipitation from testis extracts (Figure 1E; see also below), and displayed essentially complete overlap on the pericentric heterochromatin in pachytene spermatocytes (Figure 1F, top magnification) and in the chromocenters in round spermatids (data not shown). Thus, the ACF and/or CHRAC complexes form in these cells. SNF2H was also detected in spermatogonia, where BAZ1A was absent (Figure 1F, bottom magnification), consistent with presence of BAZ1A-independent ISWI complexes such as those containing SNF2H and CECR2 [39] (Figure S1A).

To provide a more detailed evaluation of chromosomal distribution during meiosis, we examined BAZ1A staining on squash preparations of spermatocyte nuclei, using anti-SYCP3 staining to evaluate stages of synaptonemal complex assembly. BAZ1A was not detectable above background until after mid-pachynema (Figure 1G), whereupon it was present diffusely on chromatin with weak enrichment in pericentric heterochromatin and strong accumulation in the sex body, a subnuclear domain containing the heterochromatinized sex chromosomes [40]. At diplonema, BAZ1A was no longer detectable in the sex body, but remained diffuse along chromatin and was now highly enriched in pericentric heterochromatin (Figure 1G). These results agree with analysis of testis sections co-stained for γH2AX, which is enriched in the sex body (Figure 1C, arrows): sex body accumulation of BAZ1A was observed in pachytene spermatocytes as early as stage V tubules (mid pachynema) and as late as stage X (late pachynema), but not diplotene spermatocytes in stage XI (Figure 1C, Figure S2B, and data not shown).

We also examined spread chromosome preparations, in which proteins are more stringently extracted from chromatin. Throughout pachynema and diplonema, BAZ1A staining displayed a punctate pattern dispersed across chromatin, with little of the enrichment in pericentric heterochromatin seen with squashes (Figure S2D). This difference suggests that BAZ1A association with pericentric heterochromatin is less robust than its binding to more dispersed sites on euchromatin. BAZ1A showed enrichment in the sex body in late pachynema, but was largely excluded there in diplonema.

### *Baz1a*-deficient mice are viable

**Figure 2:**
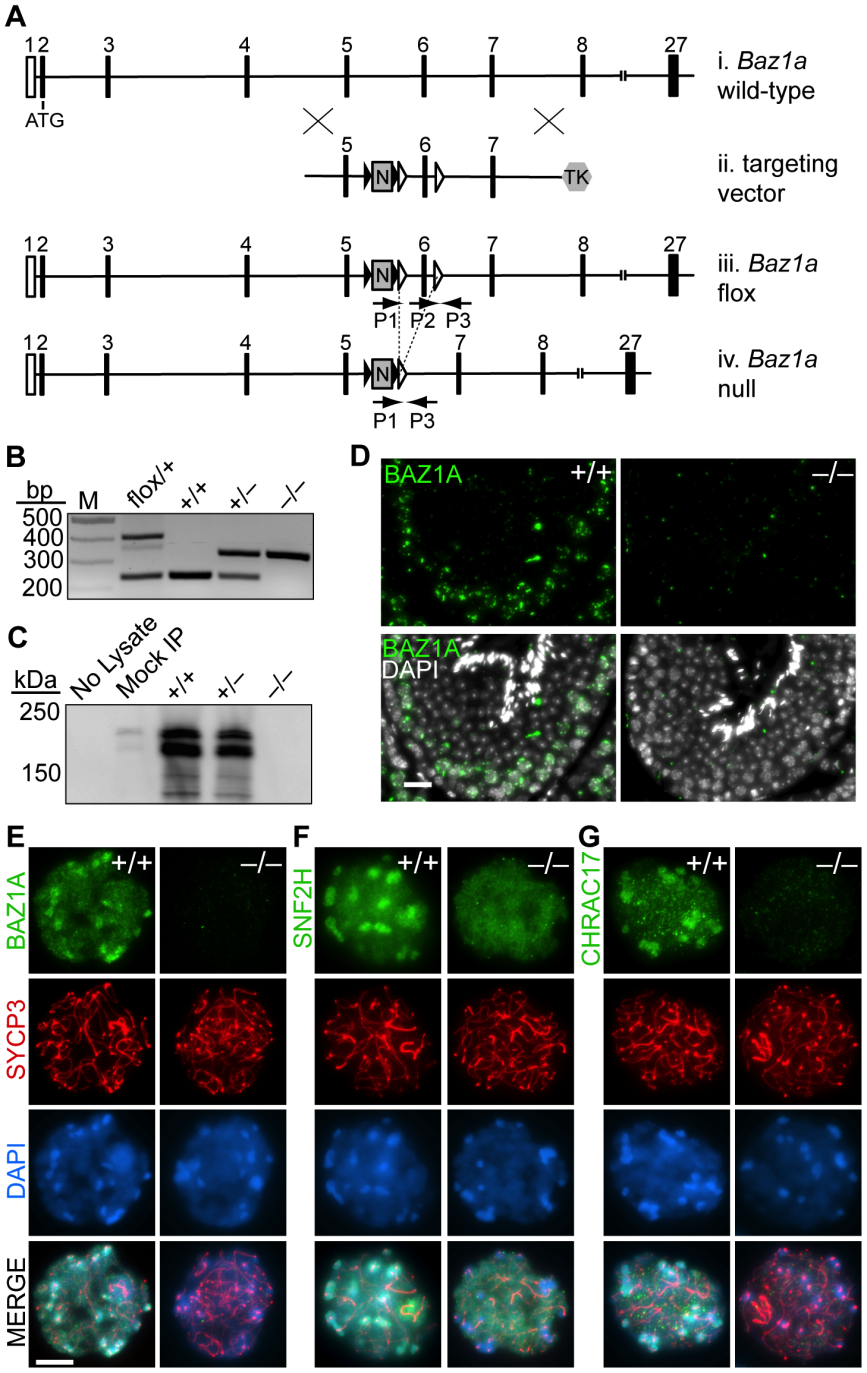
Targeted disruption of *Baz1a*. (A) Schematic of generation of *Baz1a* conditional allele. (i) Partial *Baz1a* genomic locus. (ii) Targeting vector. (iii) Conditional allele. (iv) Deletion allele following Cre-mediated recombination of the *loxP* sites (open triangles) flanking exon six. Black triangles, *FRT* sites; N, PGK-neo; TK, hsv-thymidine kinase; P1, P2 and P3, PCR primers. Not to scale. (B) PCR genotyping of tail DNA. M, marker. (C) Immunoblotting of BAZ1A immunoprecipitated from whole testis lysates. Mock IP, no antibody; No Lysate, IP from buffer. (D) Anti-BAZ1A immunofluorescence on testis sections from wild-type and mutant mice. Bar = 20 µm. (E–G) Immunoflurescence with indicated antibodies on squash preparations of diplotene spermatocyte nuclei. Bar = 10 µm.

The high level of BAZ1A expression in testicular germ cells and its dynamic localization pattern on chromatin led us to suspect that ACF and/or CHRAC might have prominent roles in spermatogenesis. To determine the *in vivo* function(s) of these complexes, we chose a conditional targeting strategy to generate a mouse *Baz1a* mutant because of the semi-lethality of a*cf1* mutation in flies [25]. *loxP* sites were inserted on either side of exon six (Figure 2A), deletion of which creates a frame shift (codon 256 of 1552 total) and introduces a premature termination codon 25 nucleotides downstream. Successful targeting in ES cells was confirmed by Southern blot (Figure S3A, B). To delete exon six, we crossed *Baz1a^flox^* mice to mice expressing Cre recombinase under the control of the male germ line-specific *Stra8* promoter [41]. Recombination of *loxP* sites was confirmed by PCR of tail DNA (Figure 2B). Males heterozygous for germ line deletion were fertile and were bred to generate mice homozygous for exon six deletion (hereafter, *Baz1a^−/−^*).

Contrary to initial expectation, *Baz1a^−/−^* mice were viable and Mendelian ratios of wild-type (95/363, 26%), heterozygous (186/363, 51%) and homozygous mutant (82/363, 23%) offspring were recovered from heterozygous crosses. Histo-pathological examination of all major organs revealed no abnormalities in *Baz1a* ^−/−^ mice (data not shown) with the exception of the testis (discussed further below).

BAZ1A protein was undetectable in *Baz1a^−/−^* animals by immunoprecipitation/western blot of whole testis extracts (Figure 2C), western blot of extracts of ear fibroblasts (EFs) derived from these mice (Figure 1B), or immunostaining of testis sections (Figure 2D) or spermatocyte squashes (Figure 2E), indicating that this is a null or severely hypomorphic allele. In wild-type diplotene cells, both SNF2H and CHRAC17 showed localization similar to BAZ1A, i.e., diffuse distribution on chromatin with enrichment in pericentric heterochromatin (Figure 2E,F,G). In *Baz1a^−/−^* spermatocytes, diffuse chromatin staining for both SNF2H and CHRAC17 was present but heterochromatin enrichment was lost (Figure 2F,G). CHRAC17 levels were also greatly reduced on *Baz1a^−/−^* chromosome spreads (Figure 2G), but total CHRAC17 levels were reduced to a lesser degree in immunoprecipitates/western blots from whole testis extracts (Figure S3C). Thus, CHRAC17 appears to be less stably bound to chromatin in the absence of BAZ1A. (Various commercial antibodies directed against CHRAC15 failed to detect a signal (data not shown)). CHRAC17 antibodies coimmunoprecipitated both BAZ1A and SNF2H from wild-type but not *Baz1a^−/−^* testis extracts (Figure S3C). These results confirm that the mutant is defective for BAZ1A function. More importantly, these findings demonstrate that BAZ1A is required for interaction between CHRAC17 and SNF2H and that a molecular function of BAZ1A is to target ACF and/or CHRAC to specific subnuclear locations.

### *Baz1a* is dispensable for the development of cells that experience programmed DSBs

RNAi directed against *BAZ1A* transcripts in cultured, tumor-derived human cells caused defects in DSB repair via non-homologous end-joining (NHEJ) and homologous recombination (HR) [31]. Moreover, GFP-tagged constructs of BAZ1A and SNF2H accumulate at sites of DNA damage in cultured cells, consistent with a possible direct role in repair of DNA damage [31], [42]. Several cell populations *in vivo* experience developmentally programmed DSBs that must be repaired for further differentiation to proceed: spermatocytes and oocytes (meiotic recombination), T-cells (V(D)J recombination) and B-cells (V(D)J and class switch recombination (CSR)). We asked whether *Baz1a* is essential in these cells.

**Figure 3:**
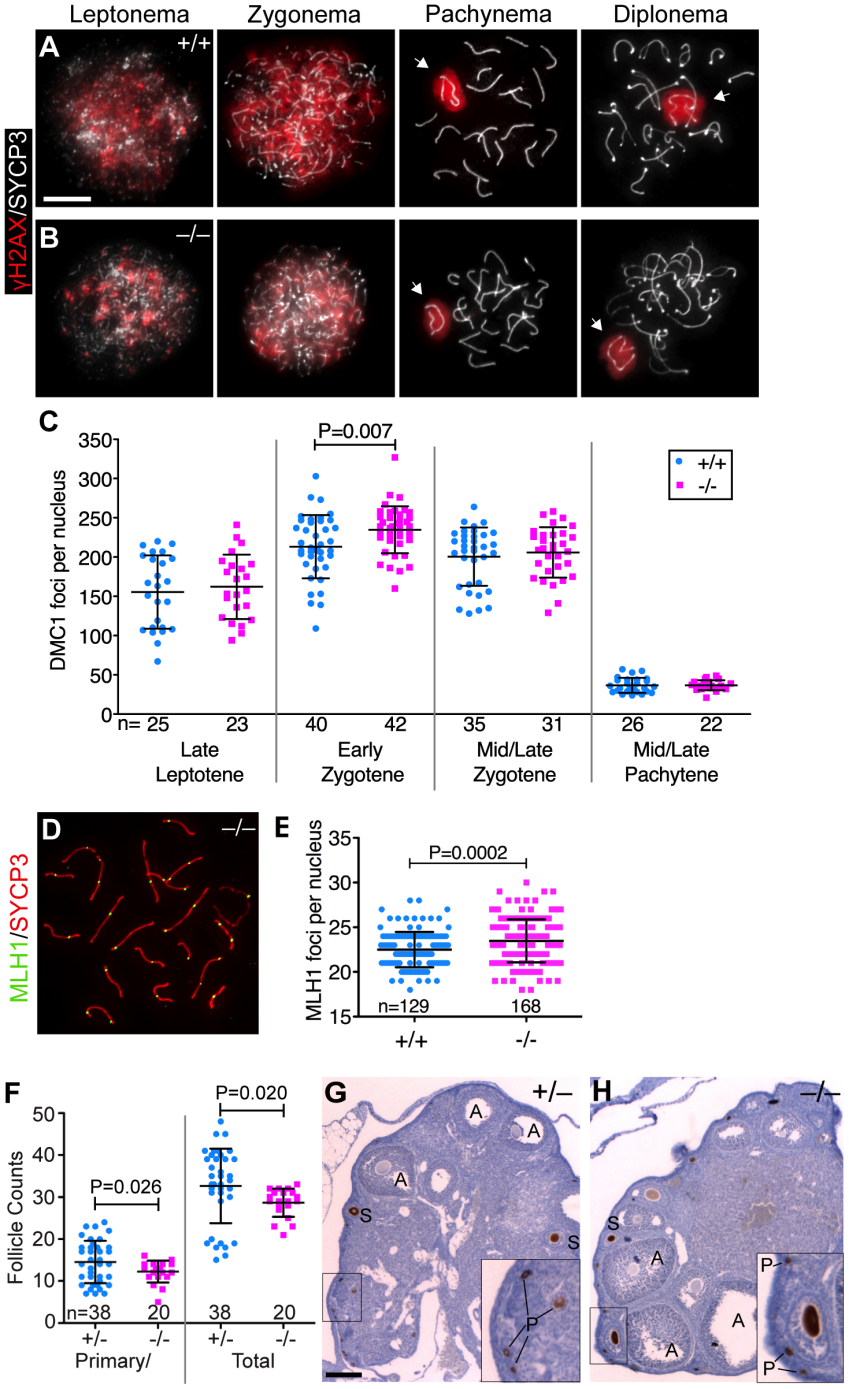
*Baz1a* mutants can repair meiotic DSBs. (A, B) Squash preparations of spermatocyte nuclei showing accumulation of γH2AX in response to meiotic DSBs, and its disappearance from autosomes as DSBs are repaired. Arrows, sex bodies; bar = 10 µm. (C) DMC1 focus counts on spread spermatocyte nuclei pooled from two sets of wild-type and mutant mice. Nuclei were staged by co-staining for the axial element protein SYCP3. Early zygotene was the only stage in which a statistically significant difference (P<0.05) was observed (P value from t-test; n = number of nuclei). (D) Spread chromosomes from *Baz1a^−/−^* pachytene spermatocyte showing MLH1 foci. (E) MLH1 focus counts pooled from two sets of wild-type and mutant littermates (P value from t-test; n = number of nuclei). (F) Primary/primordial and total follicles in *Baz1a^−/−^* and heterozygote littermate controls. Ovaries were dissected from one *Baz1a^−/−^* or two *Baz1a^+/−^* females at three months of age and stained with anti-MVH antibodies to detect oocytes. *Baz1a* ^+/−^ mice displayed no apparent phenotype in any of the cell types analyzed in this study, so they served as normal controls for these experiments. Each point is the number of follicles of the indicated type per histological section. (P values from Welch's t test; n = number of ovary sections.) (G, H) Anti-MVH-stained ovary sections from three-month old mice of the indicated genotypes. Examples at various stages of folliculogenesis are indicated: A, antral follicle; S, secondary follicle; P, primary/primordial follicle. Bar = 100 µm.

Meiotic recombination is initiated by formation of DSBs by SPO11 during leptonema [43]. Break sites are decorated by γH2AX and DMC1 foci, which dissipate as DSBs are progressively repaired during zygonema and pachynema (Figure 3A,C). In spermatocyte squash preparations, both γH2AX staining and DMC1 foci appeared and disappeared in the *Baz1a^−/−^* mutant with timing comparable to wild type, although with a slightly elevated frequency of DMC1 foci in early zygonema (Figure 3A–C). This pattern contrasts with mutants with DSB repair defects, which display persistent staining of both markers into pachynema or beyond [e.g., 44]. The large domain of γH2AX staining in both wild-type and mutant cells during pachynema and diplonema is the sex body (Figure 1C,3A,B), which appears to form normally in the mutant unlike in severely recombination-defective mutants [44]. We did not observe any impairment in synaptonemal complex formation, which is also hampered by DSB repair defects [45] (Figure 3B and data not shown). MLH1 foci, which mark nascent crossover sites, formed normally but at slightly increased frequency (Figure 3D,E). Finally, we note that mutant females were fertile (n = 4; average litter size 8.8±1.9) and had abundant oocytes in primordial and growing follicles at 3 months of age, albeit slightly reduced in number compared to littermate controls (Figure 3F–H). Follicle formation is highly sensitive to persistent SPO11-generated DSBs, such that repair-deficient mutants generally lack follicles entirely by 3–4 wks of age [46], [47]. These findings indicate that *Baz1a* is dispensable for meiotic recombination.

**Figure 4:**
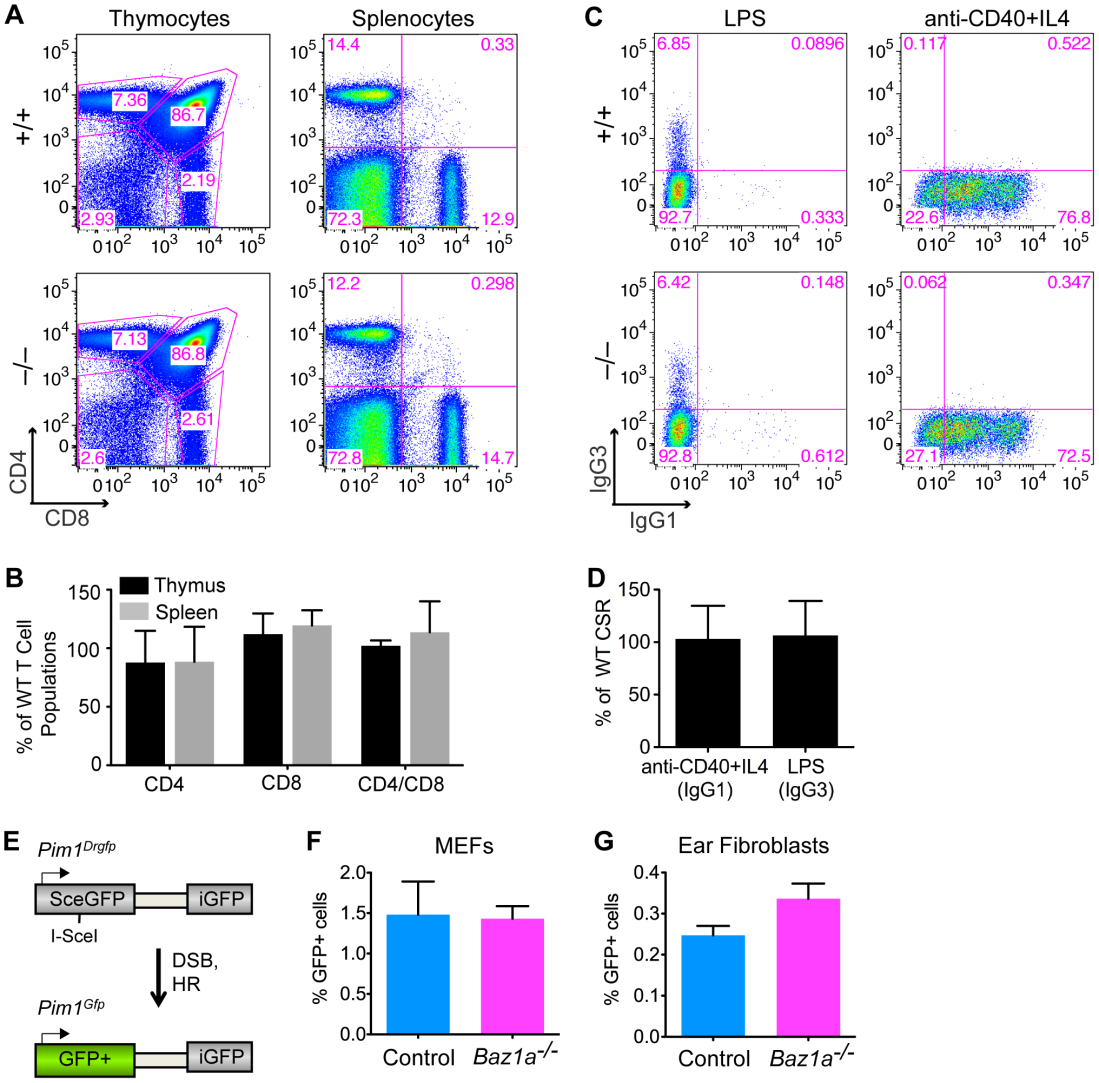
Repair of endogenously and exogenously induced DSBs in BAZ1A-deficient somatic cells. (A) Representative FACS plots of dissociated thymocytes and splenocytes expressing CD4 and CD8, implying completion of V(D)J recombination. (B) Quantification of thymocytes and splenocytes expressing CD4 and CD8 in *Baz1a^−/−^* as percent of wild-type (WT) littermates analyzed in parallel. N = 3 littermate pairs. (C) Representative FACS plots of B cells stimulated to undergo CSR to IgG1 or IgG3. (D) Cultured B cells stimulated to undergo CSR to IgG1 or IgG3 quantified in *Baz1a^−/−^* as a percentage of wild type. N = 3 littermate pairs. (E) DR-GFP reporter at the *Pim1* locus used to assay HR. Cleavage of the I-SceI site by the I-SceI endonuclease followed by repair by HR leads to GFP+ cells. (F, G) HR is not significantly different in primary cultures of MEFs (F) or ear fibroblasts (G) from *Baz1a^−/−^Pim1^+/Drgfp^* or control (*Baz1a^+/+^Pim1^+/Drgfp^* or *Baz1a^+/−^Pim1^+/Drgfp^*) mice. MEFs were derived from individual embryos from a total of two *Baz1a^−/−^Pim1^+/Drgfp^* mice and three controls. For ear fibroblasts, three independent experiments were performed using cultures derived from individual mice. Error bars in B, D, F, G are mean ± s.d.

During T cell development, RAG recombinases create DSBs to induce V(D)J recombination (rearrangement of the variable (V), diversity (D) and joining (J) gene segments of the T cell receptor (TCR) locus) to diversify the TCR repertoire [48]. Failure to repair these DSBs by NHEJ prevents normal T cell development in the thymus and reduces T cell numbers in secondary lymphoid organs, such as the spleen, which can be measured by quantifying cells expressing CD4 and CD8 co-receptors. *Baz1a^−/−^* thymus and spleen had numbers of CD4 and CD8 single- and double-positive thymocytes and T cells comparable to wild-type littermates (Figure 4A,B). Because T cells undergo selection and clonal expansion during their development in the thymus, small quantitative defects in DSB repair would not be apparent in this assay, but gross NHEJ defects can be ruled out.

In B cells, CSR is initiated by formation of DSBs in the immunoglobulin (Ig) heavy-chain locus by activation-induced deaminase (AID) and other enzymes. These DSBs are repaired by NHEJ to change the class of antibody produced [49]. Mature B cells were stimulated to undergo CSR *in vitro* by treating with anti-CD40 antibody plus IL4 to induce a switch to IgG1, or lipopolysaccharide (LPS) to induce a switch to IgG3. Indistinguishable numbers of IgG1- or IgG3-positive cells were recovered from wild-type and *Baz1a* ^−/−^ cells, indicating that CSR occurs efficiently (Figure 4C,D). Moreover, Ig variable regions are assembled from V, D and J gene segments by the same mechanisms involved in assembly of the TCR variable regions [48], and failure in this process results in developmental arrest at the CD43^+^ progenitor stage without functional Ig expression [50]. Thus, the ability to purify mature (CSR-competent, Ig^+^, CD43^−^) B cells from spleens of *Baz1a^−/−^* animals showed that V(D)J recombination is also successful during B cell development.

To further test whether BAZ1A plays a role in DSB repair by HR in somatic cells, *Baz1a* mice were crossed with mice containing the DR-GFP reporter targeted to the *Pim1* locus [51]. DR-GFP is a direct repeat of defective *GFP* genes; a DSB introduced by the I-SceI endonuclease in the upstream copy of the repeat, followed by gene conversion with the downstream copy, gives rise to GFP+ cells (Figure 4E). Early passage mouse embryonic fibroblasts (MEFs) from *Baz1a^−/−^Pim1^+/Drgfp^* embryos and control (*Baz1a^+/+^Pim1^+/Drgfp^* or *Baz1a^+/−^Pim1^+/Drgfp^*) embryos were transiently transfected with an I-SceI expression vector and analyzed by flow cytometry 48 h after transfection. No significant difference was observed in HR between *Baz1a^−/−^* and control MEFs (Figure 4F) (p = 0.885, two-tailed t test). Similarly, no significant difference in HR was observed in primary ear fibroblasts from *Baz1a^−/−^Pim1^+/Drgfp^* and control mice after transfection with the I-SceI expression vector (Figure 4G) (p = 0.110).

HR mutants, such as those defective in *Brca1*, are particularly sensitive to DNA crosslinking agents such as mitomycin C (MMC). To test for MMC sensitivity, 6 *Baz1a^−/−^* adult mice and age-matched controls were injected into the peritoneal cavity with 5 mg MMC per kg body weight. All of the *Baz1a^−/−^* mice and controls survived 21 days after treatment. By contrast, *Brca1* hypomorphic mice succumb to even lower MMC doses [51].

We conclude that in the absence of *Baz1a*, NHEJ and HR repair of developmentally programmed DSBs in the immune system and germ line, respectively, occurs relatively normally, as does HR repair of DSBs and crosslinks from exogenous sources.

### *Baz1a* mutation causes male sterility because of impaired spermiogenesis

**Figure 5:**
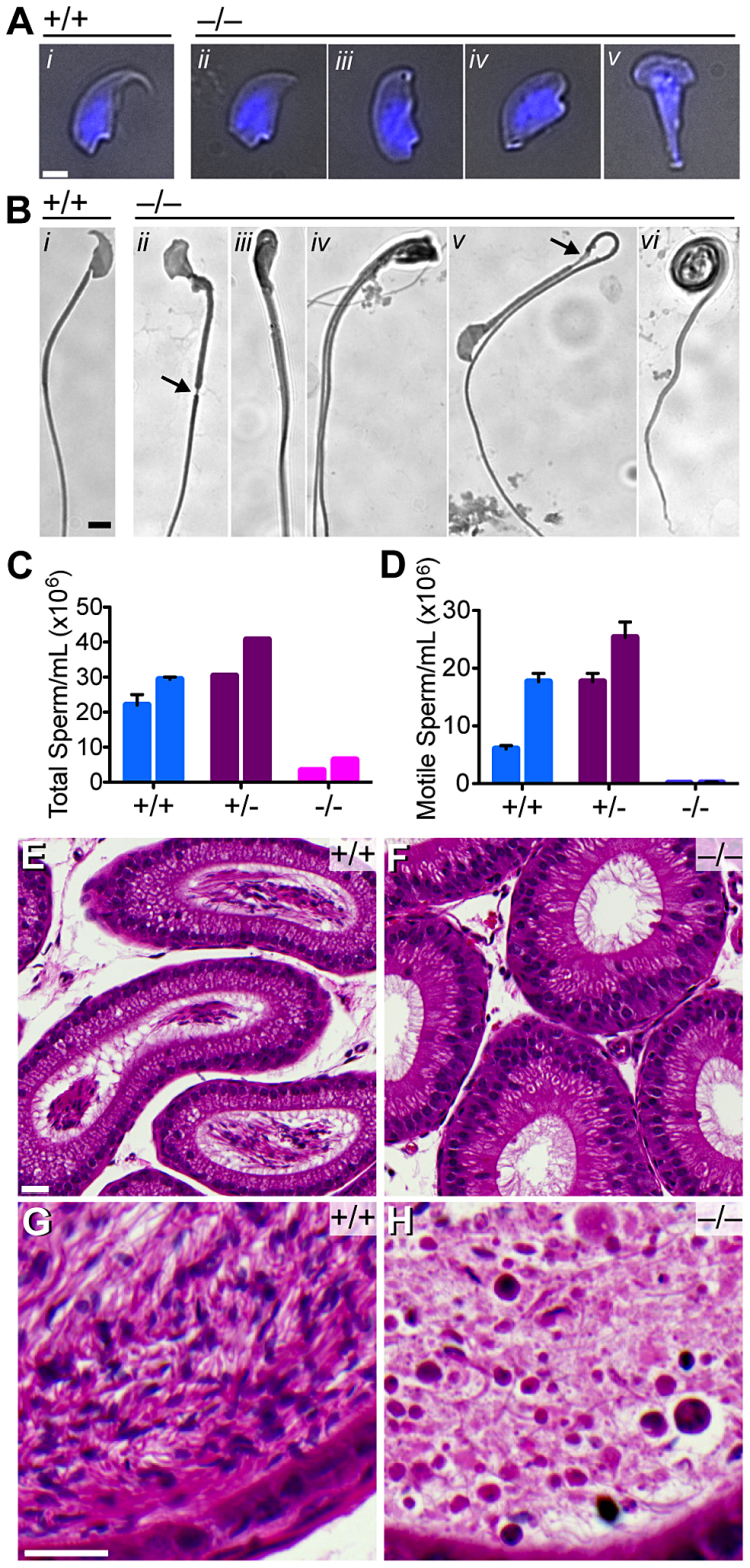
Quantitative and morphological defects of *Baz1a^−/−^* sperm. (A) Bright-field images of sperm heads overlaid with DAPI fluorescence (blue). Bar = 2 µm. (B) Bright-field images of sperm. Bar = 5 µm. (C–D) Total and motile sperm counts from total epididymides of two mice of each genotype (error bars, mean ± s.d. of triplicate counts). (E–H) Hematoxylin and eosin stained sections of caput (E, F) and cauda (G, H) epididymides. Bar = 20 µm.

Fifteen *Baz1a^−/−^* males bred with wild-type females for eight weeks produced no pups despite the presence of copulatory plugs, indicative of male infertility. Sperm from total epididymides of *Baz1a* ^−/−^ mice displayed an array of aberrant head morphologies (teratospermia), all of which lacked a normal hook characteristic of wild-type sperm heads (Figure 5A). Mutant sperm also had numerous tail abnormalities (Figure 5B) including frequent narrowing of the annulus, a domain separating the principal piece of the tail from the mid-piece (arrows in Figure 5B *ii* & *v*). Other defects included heads folded back against the tail (*iii*), two tails (*iv*), midpieces folded back against the tail (*v*), and coiling of the tail around the head (*vi*).

There was a 5-fold reduction in total sperm (oligospermia) and an almost complete absence of motile sperm (asthenospermia) (Figure 5C,D), with the exception of a few twitching movements that did not support forward progression (data not shown). Caput epididymides were devoid of sperm in the mutant in contrast to wild type, which displayed lumens packed with mature sperm (Figure 5E,F). Unlike wild-type cauda epididymides, where darkly stained sperm heads and tangles of lightly stained tails were visible (Figure 5G), those from the mutant contained only debris and degenerating, mostly round cells that likely sloughed from the seminiferous tubules (Figure 5H).

Injection of nuclei from *Baz1a^−/−^* round spermatids isolated from testes into wild-type oocytes, followed by artificial activation, supported formation of embryos competent to reach at least the blastocyst stage (6 of 19 successful injections, 32%). In contrast, intracytoplasmic sperm injection (ICSI) using *Baz1a^−/−^* epididymal sperm failed to spontaneously activate oocytes, and even after artificial activation, only rarely yielded embryos reaching the blastocyst stage (1 of 23 successful injections). In a control experiment using sperm from unrelated wild-type mice, 21 of 47 successfully injected embryos (45%) developed to the blastocyst stage. Therefore, whereas *Baz1a^−/−^* round spermatids are competent to support early embryonic development, mutant sperm are compromised in both oocyte activation and preimplantation development.

**Figure 6:**
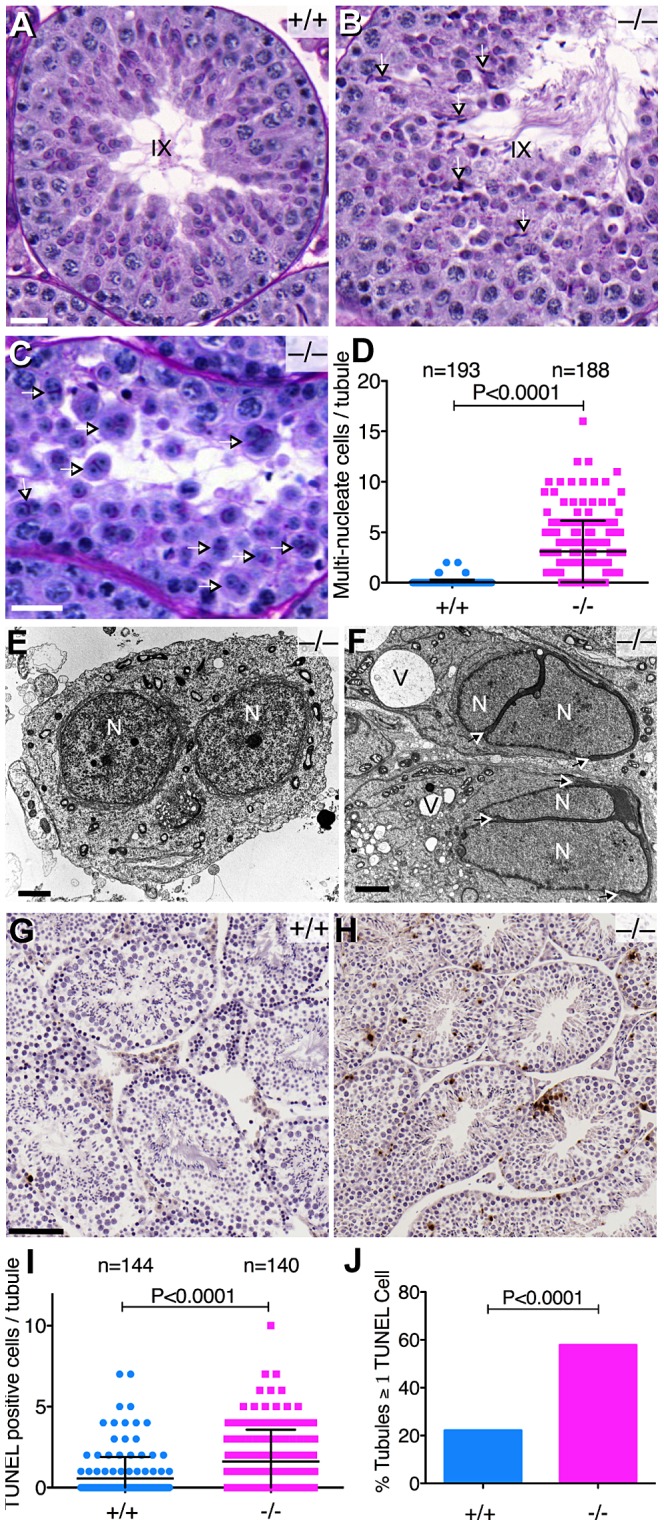
Abnormal spermatogenesis in the absence of *Baz1a*. (A, B) Periodic Acid-Schiff and hematoxylin stained sections of stage IX seminiferous tubules showing sperm retention (arrows) in the mutant. Bar = 20 µm. (C) Mutant tubule with multi-nucleate round spermatids (arrows). Bar = 20 µm. (D) Quantification of multi-nucleate cells (error bars, mean ± s.d.). (E, F) Transmission electron microscopy of mutant testis sections showing a multi-nucleate round spermatid (E) and elongating spermatids (F). Arrows indicate the degree of acrosome stretching. N, nucleus; V, vacuole; bar = 2 µm. (G, H) TUNEL staining of testis sections. Bar = 100 µm. (I, J) Quantification of TUNEL-positive cells per tubule (I) (p value from t-test) and percentage of tubules with ≥1 TUNEL-positive cell per tubule (J) (p value from Fisher's exact test). Error bars, mean ± s.d.

Seminiferous tubule sections showed multiple defects in *Baz1a* mutants. Some stage IX tubules displayed spermiation failure as indicated by the presence of mature sperm, which should have been fully released into the lumen at this stage (compare Figure 6A and B). There was also a high frequency of multi-nucleate cells in tubules of various stages (Figure 6C,D). Electron microscopy on testis sections revealed frequent bi-nucleate round spermatids (Figure 6E) and bi-nucleate elongating spermatids, often with a single acrosome stretching over both nuclei (Figure 6F). Thus, the highly ordered spermiogenic program is thrown off course in the absence of *Baz1a*, resulting in a range of aberrations.

Testis sections from *Baz1a^−/−^* mice also displayed a modest but significant increase in the average number of apoptotic cells per tubule and a three-fold increase in the percentage of mutant tubule sections with one or more apoptotic cells (Figure 6G–J). Apoptosis did not appear to be restricted to any particular cell type because TUNEL-positive cells were found scattered throughout tubule sections irrespective of stage (Figure 6H and data not shown). Moreover, apoptosis was too infrequent to account quantitatively for the decrease in mature spermatozoa or the morphological defects.

### Gross changes in chromatin protein composition occur on schedule during *Baz1a*-deficient spermatogenesis

**Figure 7:**
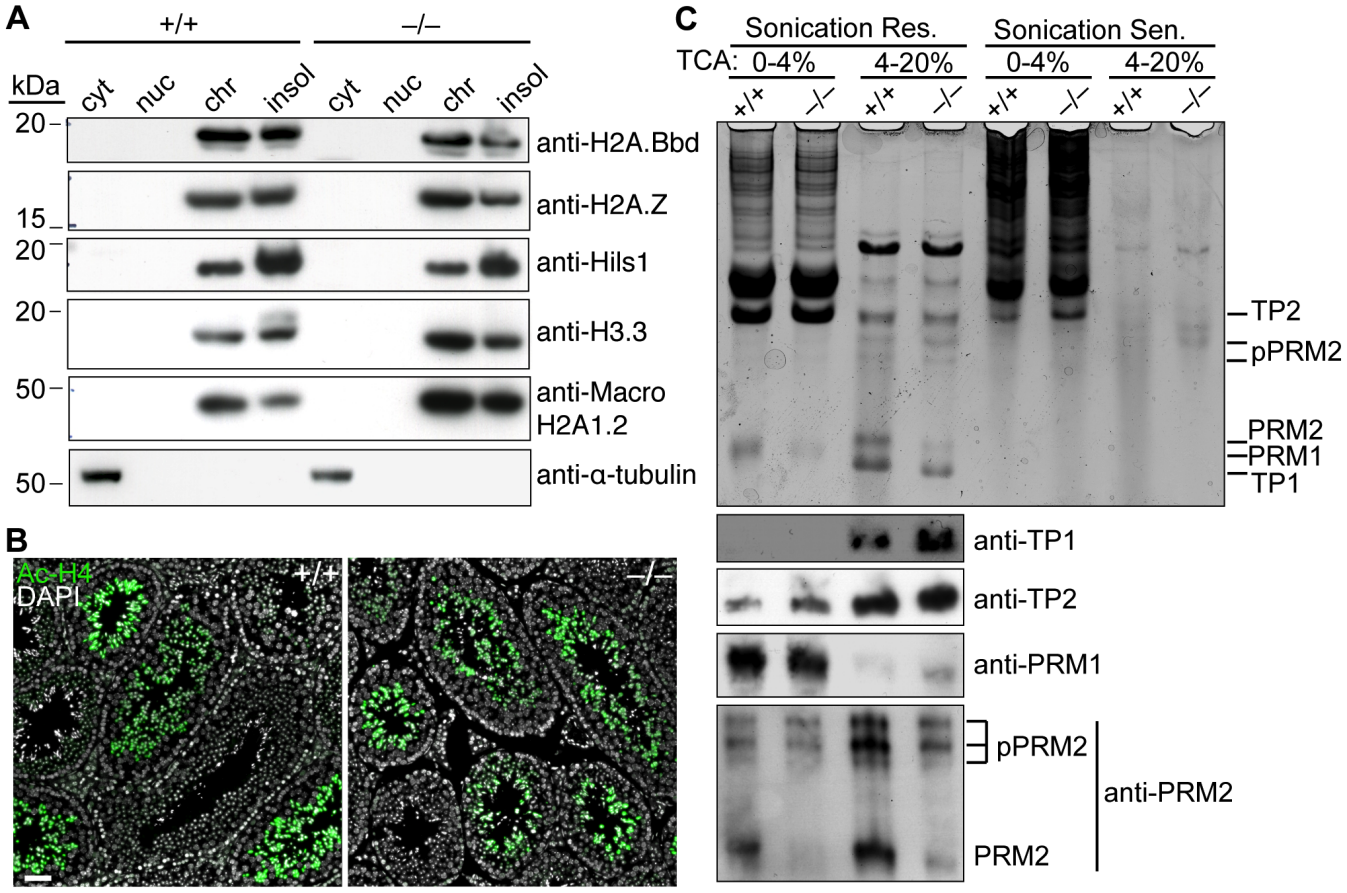
*Baz1a* is dispensable for spermatogenesis-associated changes in chromatin protein composition. (A) Testis cells were fractionated into cytoplasmic (cyt), nucleoplasmic (nuc), chromatin bound (chr) and insoluble (insol) extracts and immunoblotted for a panel of testis-specific histone variants. α-tubulin is a cytoplasmic marker. (B) Immunofluorescence on testis sections with anti-acetyl histone H4 (Ac-H4) antibody. Bar = 50 µm. (C) HCl-extracted proteins from sonication resistant (Res.) and sensitive (Sen.) spermatids were precipitated with 4% and then 20% trichloroacetic acid (TCA), separated by acid/urea PAGE and stained with Coomassie (top panel). A duplicate gel was used for western blotting with antibodies against the indicated proteins (bottom panels).

Since BAZ1A is a chromatin-remodeling factor, we considered that it may be required for the large-scale replacement of chromatin proteins that occurs during spermatogenesis [33]. To test this, we looked at the exchange of somatic histones for testis-specific histone variants by sub-cellular fractionation of whole testes followed by immunoblotting for various histone variants. All of the variants examined were expressed and present in the chromatin-enriched fraction (Figure 7A). These findings indicate that *Baz1a*-deficiency does not globally disrupt this process, although we cannot rule out the possibility that other histone variants are improperly loaded, and these experiments do not address whether the spatial organization of the replaced histones is normal. Following incorporation of histone variants, N-terminal lysines of histone H4 are hyper-acetylated, which is thought to aid in the histone-to-protamine exchange that follows [52]. Immunostaining for pan-acetyl-H4 showed comparable signal in the lumen-proximal elongating spermatids from both genotypes (Figure 7B), suggesting hyper-acetylation was also unperturbed in the mutant.

The highly basic transition proteins (TP1 and 2) and protamines (PRM1 and 2) that replace histones were extracted from late-stage spermatids, which are sonication-resistant because of their compact nature. Coomassie staining of acid-urea polyacrylamide gels revealed bands with the expected migration of all four proteins and the higher molecular weight precursors of PRM2 (pPRM2), which are proteolytically cleaved to yield the mature form (Figure 7C, top panel). PRM1 is less soluble in TCA and was therefore detected in the 0–4% cut while the other proteins were detected in the 4–20% cut. As expected, these bands were largely undetectable in the sonication-sensitive extracts. Immunoblotting confirmed the identity of the bands (Figure 7C, bottom panels). Levels of mature PRM2 were modestly decreased in sonication-resistant mutant spermatids (Figure 7C, bottom panels). However, similar reductions have been observed in other spermiogenesis mutants [53]–[55], so this may be a nonspecific consequence of developmental arrest rather than a causal factor.

Taken together, these findings indicate that BAZ1A is dispensable for global spermatogenesis-associated changes in chromatin protein composition. This is consistent with findings in *Drosophila*, where Acf1 is dispensable for nucleosome deposition per se [25]. However, whereas Acf1 promotes heterochromatinization in flies [26], [56], we found that *Baz1a*-deficient cells display normal staining patterns of heterochromatin markers HP1β, HP1γ and H3K9Me3 in primary spermatocytes and round spermatids (Figure S4), suggesting BAZ1A is not needed for heterochromatin formation in mouse.

### *Baz1a^−/−^* spermatids display widespread RNA mis-expression

*Baz1a* has been implicated in transcriptional regulation in other organisms [27], [30], so we hypothesized that altered gene expression might be the cause of defective spermiogenesis. We therefore compared RNA profiles of pachytene/diplotene spermatocytes and round spermatids from *Baz1a^−/−^* and heterozygous littermates, using cells highly enriched by FACS of dissociated testes (Table S1). *Baz1a^+/−^* mice displayed no overt phenotype, so they served as normal controls for these experiments.

**Figure 8:**
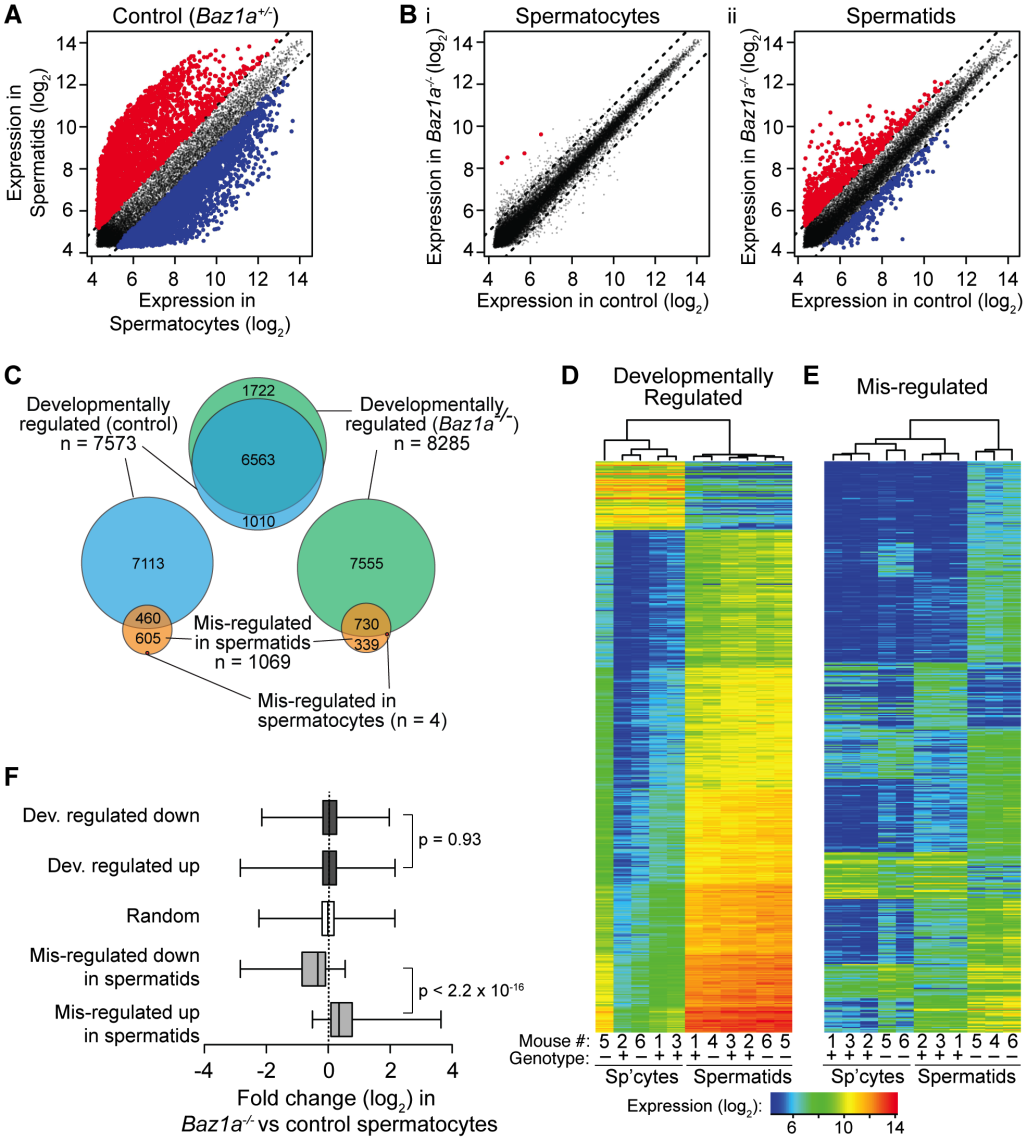
Wide-spread transcriptional perturbations in *Baz1a*-deficient spermatids. (A) Differential expression in normal spermatids vs. spermatocytes. Each point plots expression of a microarray probe averaged between samples. Red, significantly up regulated in spermatids (≥2-fold, q = 0.05); blue, significantly down regulated; gray, not significantly different. Dashed lines indicate two-fold change. (B) Differential expression between *Baz1a^−/−^* and control spermatocytes (i) or spermatids (ii). Plotting and color code as in A. (C) Proportional area Venn diagrams showing overlap of differentially expressed probe sets. “Developmentally regulated” probes were differentially expressed in spermatids compared to spermatocytes (≥2-fold, q = 0.05); “mis-regulated” probes were differentially expressed in *Baz1a^−/−^* vs. control. (D, E) Heat maps of expression levels in all samples of developmentally regulated and mis-regulated probes. The 500 most differentially expressed probes were chosen from comparison of spermatids vs. spermatocytes in controls (“developmentally regulated”, D) or *Baz1a^−/−^* vs. control spermatids (“mis-regulated”, E). Samples were clustered hierarchically and probes were grouped by k-means clustering (k of 7 and 10 for D and E, respectively). Mouse number and *Baz1a* genotype of samples are indicated (+ = control; − = *Baz1a^−/−^*). (F) Box and whisker plots of expression changes in *Baz1a^−/−^* vs. control spermatocytes for the indicated probe sets. Boxes show median and interquartile range, whiskers extend from minimum to maximum values; p values are from Wilcoxon rank sum tests.

Prior studies demonstrated large-scale expression changes accompanying the developmental switch from meiosis to postmeiotic differentiation [57], [58]. Accordingly, we found that 7573 microarray probes representing 5656 genes showed at least two-fold change in expression when normal control spermatocytes and round spermatids were compared (false discovery rate (FDR) q = 0.05) (Figure 8A and Table S2). This accounts for 38.2% of all probes detectably expressed in at least one of the samples analyzed. The probes with greatest change in expression were highly enriched for up-regulation in spermatids (83.2% of the top 1000 probes) (Figure 8A). Thus, much of the postmeiotic gene expression program involves transcriptional activation of previously quiescent genes.

*Baz1a^−/−^* spermatocytes were grossly similar to controls using standard criteria for defining differential expression: only four probes scored as significantly different (≥2-fold, q = 0.05), all higher in the mutant (Figure 8B *i* and Table S2). In contrast, round spermatids displayed widespread changes: 1069 probes (912 genes; 5.4% of expressed probes) were altered two-fold or more in the mutant (Figure 8B *ii* and Table S2), of which 70.3% were higher in *Baz1a^−/−^*. Thirty-six probes differed by ≥8-fold, all but one higher in the mutant. Thus, substantial disruption of gene expression accompanies progression from meiosis into spermiogenesis in the absence of BAZ1A, most often reflecting aberrantly high expression. If the multi-nucleate spermatids frequently observed in the mutant represent a subset of cells with more severe transcription defects, it is possible that this analysis underestimates the degree of mis-regulation. These cells would be largely absent from the haploid round spermatid population enriched by FACS.

We asked how mis-regulation in *Baz1a* ^−/−^ cells compares with the expression changes across the normal spermatocyte-to-spermatid transition. Multiple lines of evidence show that BAZ1A deficiency affects a largely independent group of transcripts, leaving developmentally programmed expression changes essentially intact. First, there was little overlap of the two probe sets beyond that expected by chance. Of 1069 mis-regulated probes, 460 also scored as developmentally regulated in controls (43%; Figure 8C). This overlap is only slightly above the fraction expected from two random samples (p = 0.04, Fisher's exact test), because the developmental set is so large (38.2% of expressed probes). More importantly, only 39 mis-regulated probes (3.6%) overlapped with the top 1000 developmentally regulated probes, significantly less than the 5.1% overlap expected by chance (p = 0.043). Additionally, the probes found on both lists showed poor correlation with respect to the magnitude and direction of expression changes observed (data not shown).

Second, hierarchical clustering based on developmentally regulated probes clearly distinguished samples by cell type as expected, but did a poor job of stratifying spermatocyte or spermatid samples by genotype (Figure 8D). In contrast, clustering based on the mis-regulated probes clearly separated control from mutant samples (Figure 8E). Thus, sample-to-sample variation is greater than variation by genotype for developmentally regulated probes, i.e., BAZ1A deficiency has relatively little impact on expression of these probes' targets.

Third, the large expression changes that accompanied the spermatocyte-to-spermatid transition overlapped extensively between *Baz1a^−/−^* animals and controls (Figure 8C). More of these developmentally regulated probes scored as differentially expressed in the mutant (8205 probes, 6203 genes; Table S2), which overlapped with 86.7% of the developmentally regulated probes in controls and 68.3% of mis-regulated probes in *Baz1a^−/−^* spermatids (Figure 8C). We infer that mutant spermatids amalgamate two separate patterns: normal postmeiotic expression changes plus distinct defects caused by BAZ1A deficiency.

Fourth, *Baz1a^−/−^* cells showed normal sex chromosome expression. Most genes on X and Y are silenced in prophase by meiotic sex chromosome inactivation (MSCI), but a subset are reactivated or induced de novo in spermatids [57], [59]. Consistent with prior findings, 19.9% of X and Y probes were up-regulated during the transition from spermatocyte to spermatid in controls, including essentially all of the previously identified genes present on the array (Figure S5A and data not shown). Indeed, developmentally regulated probes were significantly overrepresented on X and Y compared to autosomes (Figure S5C and data not shown). *Baz1a^−/−^* mutants had nearly indistinguishable expression for X and Y probes in both cell types (Figure S5B), such that these probes were greatly underrepresented in the mis-regulated class (Figure S5C). Thus, both MSCI and postmeiotic activation of X and Y genes are independent of BAZ1A.

As noted earlier, many histone variants and histone posttranslational modifications, transition proteins, and protamines were expressed normally. Moreover, expression and loading of MIWI-bound piRNAs was normal in the absence of *Baz1a* (Figure S6A) and expression of several repetitive elements was unchanged or slightly decreased (Figure S6B), indicating that these processes are also regulated appropriately. We deduce that much of the altered gene expression in *Baz1a^−/−^* reflects mis-regulation of a distinct set of genes overlaid on the normal developmental program.

### Altered gene expression is also visible in mutant spermatocytes, but to a lower degree

When does mis-regulation begin? Although few probes scored as significantly different between control and *Baz1a^−/−^* spermatocytes, mutant spermatocytes did show evidence of the aberrant pattern seen more strongly in spermatids. Specifically, hierarchical clustering based on the most mis-regulated probes in spermatids also separated spermatocyte samples by genotype (Figure 8E). Several probe clusters appeared to have parallel patterns of up- and down-regulation in both cell types, and the four probes scored as differentially expressed in spermatocytes were in the top 1.8% of probes mis-regulated in spermatids (Table S2). From this, we suspected that mis-regulation had already begun to manifest in meiotic prophase I.

In support of this conclusion, differential expression in spermatocytes correlated strongly with that in spermatids even though most individual probes did not score as significantly altered using standard cutoffs for fold change and statistical significance. Specifically, most probes that were inappropriately up-regulated in mutant spermatids were also elevated in *Baz1a^−/−^* spermatocytes and, conversely, most probes down-regulated in mutant spermatids were also lower in mutant spermatocytes (1.4-fold on average for both sets) (Figure 8F). Randomly chosen probes showed no enrichment one way or the other, as expected (Figure 8F).

Whereas highly pure round spermatid populations were readily obtained by FACS, spermatocytes showed greater sample-to-sample variability (Table S1). In principle, the appearance of differential expression in spermatocytes could arise from spermatid contamination, but several lines of evidence rule this out. First, the four probes significantly up in mutant spermatocytes were not among those up-regulated during the spermatocyte-to-spermatid transition in controls (Table S2). Second, *Baz1a^−/−^* and wild-type spermatocytes showed no significant difference when developmentally up-regulated and down-regulated probes were compared; indeed, the expression distribution for these probe sets was similar to that of a random probe selection (Figure 8F). Finally, as noted above, hierarchical clustering based on developmentally regulated probes did not stratify spermatocyte samples by genotype, although signatures of variable spermatid contamination can be seen (Figure 8D). Notably, mouse 6 yielded the purest of the *Baz1a^−/−^* spermatocyte populations based on microscopic inspection (Table S1), depletion of spermatid expression signature for developmentally regulated probes (Figure 8D), and low expression of X and Y probes (data not shown). This sample grouped with controls when clustered on developmentally regulated probes (Figure 8D), but grouped with the other *Baz1a^−/−^* spermatocyte sample (mouse 5) when clustered on mis-regulated probes (Figure 8E). These observations are not consistent with spermatid-derived RNA contributing significantly to genotype-correlated expression differences in spermatocyte samples.

### Characteristics of genes mis-regulated in the absence of BAZ1A

The genes mis-regulated in *Baz1a^−/−^* spermatogenesis are heterogeneous with a wide range of annotated functions. Based on enrichment for gene ontology (GO) terms, prominent categories among inappropriately up-regulated genes included peptidases (30 genes, adjusted p value for enrichment of 0.009), transcription factors (37 genes, p = 0.012), cytoskeleton proteins (35 genes, p = 0.51), and cell-cell junction proteins (10 genes, p = 0.34). Genes in these categories that were up ≥8-fold in mutant spermatids included those for serine proteases CAPN9 and kallikreins (KLK1b21, 24, and 27); transcription factors SP5, SPDEF, IRF1, LHX1, and TCF7L1; myosin MYO1F and ankyrin ANK1; and integral membrane proteins ABCB4 and CLDN5 (Table S2).

We found no obvious properties linking these disparate genes: they are distributed widely among all autosomes (Figure S5C) without evidence of local clustering (data not shown), and we were unable to identify DNA sequence motifs or annotated transcription factor binding sites that are specifically enriched in up-regulated promoters (data not shown). Of note, 25% of up-regulated probes were not detectably expressed in wild-type spermatids, and 20.1% of down-regulated probes were not detectable in the mutant. Thus, many genes expressed in the mutant should not be expressed at all, and many suppressed genes should be transcribed.

## Discussion

ACF and CHRAC were first isolated from *Drosophila* extracts as activities that promote assembly of phased nucleosomes *in vitro*. Since then, extensive biochemical, biophysical, and structural analyses, particularly of human and *Drosophila* ACF and the yeast ortholog ISW2, have delved deeply into the mechanisms by which these and other ISWI complexes operate. Their functions *in vivo* have been less well characterized, however, particularly in mammals. We addressed these questions here by analysis of the expression and localization of BAZ1A, a defining subunit of ACF and CHRAC, and by characterization of mice homozygous for a targeted *Baz1a* mutation.

### BAZ1A and DSB repair

Recent reports implicated BAZ1A in DSB repair in transformed human cell lines [31], [32]. Numerous other chromatin remodeling complexes play roles in DSB repair and/or DNA damage responses, including the large multi-protein chromatin remodeling complexes of the SWI/SNF and INO80 families [60] and the ISWI family member WICH (SNF2H in complex with BAZ1B) [61]. Thus, it was plausible that ACF/CHRAC might fit into this growing list. However, *Baz1a*-deficient mice showed no signs of substantial DSB repair defects in cell populations where developmentally programmed DSBs occur—spermatocytes, oocytes, T-cells, and B-cells. Moreover, I-SceI-generated DSBs induced similar HR frequencies in the presence and absence of BAZ1A in two different somatic cell types, and *Baz1a^−/−^* mice did not display overt sensitivity to MMC exposure *in vivo*, unlike known HR-defective mutants. We conclude that BAZ1A (and by extension ACF and CHRAC) are largely if not completely dispensable for DSB repair in mouse cells.

One possibility to explain the apparent difference between human cells *in vitro* and mouse cells *in vivo* could be species-specific differences in the expression of BAZ1A. We note, however, that immunoblotting demonstrated comparable protein levels in primary and tumor-derived mouse cells and the human cell lines (HeLa and U2OS) used for prior RNAi studies (Figure 1B). (Note that the antibody was raised against a 135 amino acid epitope of human BAZ1A which only shares 85% peptide sequence identity with mouse BAZ1A, so if anything this analysis may slightly underestimate relative levels of the mouse protein.) Another possibility is that DSB repair defects could be tied to presence of other mutations that sensitize the tumor-derived human cell lines studied to BAZ1A depletion. Moreover, off-target effects of the RNAi experiments cannot be ruled out as complementation of the cell lines with RNAi-resistant *BAZ1A* was not conducted. Other differences in methodology may also contribute, namely acute depletion by RNAi vs. genetic ablation. *Baz1a* ^−/−^ mutants and cells derived from them will provide ideal tools to address these issues.

### BAZ1A deficiency causes a unique gene mis-regulation signature in spermatogenesis

We show here that mouse BAZ1A, and by inference ACF and/or CHRAC complexes, are essential for spermiogenesis and male fertility. Male *Baz1a^−/−^* germ cells displayed widespread mis-regulation of gene expression beginning at least as early as meiotic prophase and growing substantially worse during early postmeiotic differentiation. By and large, the normal transcription program was executed correctly and in a timely manner.

This phenotype is distinct from that of mutations such as those affecting the A-MYB or CREM transcription factors, which derail the normal meiotic and/or postmeiotic expression programs primarily because of failure to properly up-regulate target genes [37], [62], [63]. Specifically, CREM and A-MYB regulated genes were not enriched in the list of misregulated genes in *Baz1a^−/−^* (data not shown), and protein expression of several known CREM targets was normal (i.e., protamines and transition proteins; Figure 7C). Failed transcription activation in mutants lacking these key transcription factors contrasts with the widespread derepression in the absence of BAZ1A.

More importantly, the *Baz1a^−/−^* gene expression signature is wholly unlike that of other chromatin modifying factor mutants. For example, BRDT is a bromodomain-containing protein that binds acetylated histone H4 and is expressed from pachynema through round spermatid stages (i.e., substantially overlapping with BAZ1A expression) [64]. Genetic ablation or chemical inhibition of BRDT causes male sterility because of spermatogenic failure tied to defects in inducing the normal spermatogenic transcription program [35], [65]. RNF8 is a ubiquitin E3 ligase that ubiquitylates histone H2A [66]; unlike *Baz1a^−/−^*, *Rnf8^−/−^* mutants specifically fail to reactivate silenced X and Y genes in round spermatids [67]. BRG1 is a subunit of BAF, a chromatin remodeling complex of the SWI/SNF family (distinct from the ISWI family). *Brg1*-deficient spermatocytes arrest in prophase because of recombination defects [68], [69]. Finally and most relevant to our studies, CECR2 is a BAZ1A paralog that forms complexes with ATPases SNF2H and SNF2L (Figure S1A, S1C) [39], [70]. However, in striking contrast to BAZ1A-deficient animals, *Cecr2^−/−^* mutants have only modestly reduced male fertility because of a defect in the ability of spermatozoa to fertilize oocytes, show no gross defects in spermatogenesis or sperm number and motility, and have only minor changes in testis gene expression (35% change in one gene of 12 tested) [39].

### Function of BAZ1A in spermatogenic gene regulation

Although the gene mis-regulation signature in *Baz1a^−/−^* spermatocytes is unlike that in other described chromatin mutants in mouse, it is entirely congruent (albeit it in a different cellular context) with a common thread connecting phenotypes of yeast and fly *acf* mutants, namely defects in transcriptional control largely because of inappropriate derepression. Specifically, *acf1* mutant flies have defects in forming Polycomb-dependent repressive chromatin and repressing Wingless-regulated genes [26], [27], and yeast mutants lacking ISW2 complexes derepress numerous genes [71], [72]. Similarly, RNAi experiments implicate human BAZ1A in repressing vitamin D3 receptor-regulated genes as well [30]. In keeping with known biochemical properties of ACF/CHRAC (see Introduction), the influence of these remodelers on gene expression in these diverse taxa is either known or hypothesized to be via direct control of chromatin structure [73]. It is therefore parsimonious to infer that the same is likely true for BAZ1A in spermatogenesis. Importantly, while targeted studies have identified a handful of ACF-regulated genes in flies and human cells, ours is the first to address this question genome-wide.

Importantly, we note that mis-regulation in *Baz1a^−/−^* coincides with the dramatic chromatin changes that accompany spermatogenesis and that occur on time in the mutant, e.g., exchange of canonical and variant histones. We also note that BAZ1A first becomes abundant on chromatin in pachynema, the time mis-regulation first begins to be detected in the mutant. This places BAZ1A on chromatin at the right time and place to be directly involved in controlling gene expression by modulating chromatin structure. We hypothesize that the shifting chromatin protein composition renders the genome vulnerable to promiscuous transcription changes, and that an essential function of ACF and/or CHRAC is to safeguard against these changes by ensuring that newly assembled chromatin is structured correctly. In this view, many of the genes observed to be mis-regulated may be direct targets of BAZ1A-dependent chromatin remodeling, but indirect effects are also likely to be at play, e.g., as a downstream consequence of inappropriate expression of transcription factors or other genes. Interestingly, although mis-expression was widespread, it was not universal, thus not all genes are equally vulnerable.

### The cause of spermatogenic failure in *Baz1a^−/−^* males

*Baz1a*-deficient mice showed highly pleiotropic defects in spermatid development. Importantly, mis-regulation in the mutant had already begun by late prophase, i.e., well before overt cellular defects were apparent, suggesting that transcription defects tied directly to absence of ACF and/or CHRAC are unlikely to be a consequence of spermiogenic failure. Many of the *Baz1a^−/−^* differentiation defects (e.g., multinucleate round spermatids, non-motile sperm, aberrant head and tail morphologies) have been described in numerous other mutants affecting distinct molecular pathways [74]. In other words, different molecular defects can give similar phenotypic endpoints, so the precise ways in which spermiogenesis goes awry are not uniquely diagnostic. Not surprisingly then, the eclectic collection of genes mis-regulated in the mutant provided no single smoking gun to explain the cellular phenotypes, so a simple explanation is that the massive mis-regulation per se is the proximal cause of sperm differentiation defects. Indeed, we favor the view that *Baz1a^−/−^* pleiotropy reflects the large number of mis-expressed genes, the variety of their functions, and the all-or-nothing nature of some mis-regulation. Importantly, our data conclusively rule out alternatives that could otherwise have been considered possible causes of infertility, e.g., defects in piRNA production, control of transposable elements, or regulation of sex chromosome gene expression.

### Other possible functions of BAZ1A

BAZ1A displayed dynamic subnuclear localization patterns during meiosis and spermiogenesis. Because mis-regulated genes were scattered among all autosomes without obvious clustering, we consider it likely that BAZ1A-dependent enforcement of appropriate gene expression is related to the diffuse euchromatic staining that was relatively resistant to extraction in chromosome spreads during pachynema and diplonema. However, other prominent staining patterns are not obviously tied to essential functions of the protein. For example, BAZ1A was transiently enriched in the sex body during late pachynema, but *Baz1a* ^−/−^ cells displayed no changes in either MSCI or postmeiotic activation of XY genes. If ACF/CHRAC contribute to proper function of the sex body, they are either redundant with other factors or are involved in processes that are dispensable for proper control of sex chromosome gene expression. Similarly, ACF and/or CHRAC are highly enriched in pericentric heterochromatin in cultured cells [29], [38] and during spermatogenesis (this study), but no gross heterochromatin defects were observed in *Baz1a*-deficient spermatocytes or round spermatids. Furthermore, we did not observe fragmentation of the heterochromatic chromocenter such as that reported for *Brdt*-deficient spermatids [75], and the *Baz1a^−/−^* mutant does not phenocopy mutants known to have heterochromatin defects such as those with deficiencies in the histone methyltransferase SUV39h, the Polycomb-group transcription factor YY1, or HP1γ [76]–[78]. The function of this binding to heterochromatin is thus unclear, and is in contrast with findings in *Drosophila*, where *acf1* mutants are deficient for accumulating heterochromatic marks equivalent to those assayed here [26]. Intriguingly, BAZ1A became undetectable as spermatids began to elongate, coincident with replacement of histones by transition proteins, but then reappeared later, at about the time when protamines replace transition proteins. This behavior may reflect additional roles for BAZ1A (and ACF/CHRAC) in late-spermiogenesis chromatin remodeling.

BAZ1B (also known as WSTF) is the closest paralog of BAZ1A by sequence and also associates with SNF2H to form the WICH complex (Figure S1A, S1C). While not up-regulated in testis, BAZ1B is present, raising the possibility that it may have distinct or possibly overlapping functions with BAZ1A. *Baz1b* mutation causes perinatal lethality because of cardiac defects [79], so a conditional targeting strategy would be necessary to test for function in the germ line.

Interestingly, human *BAZ1A* was down-regulated in testis tissue displaying round spermatid maturation arrest, isolated from infertile men with azoospermia [80]. Thus, it is possible that decreased BAZ1A levels may also lead to human male sterility. Moreover, the only clearly essential function revealed by our study is in support of spermiogenesis and male fertility, suggesting that small-molecule inhibitors of BAZ1A could confer male contraceptive activity analogous to that shown recently for inhibitors of BRDT [65].

## Materials and Methods

Ethics statement: All animal work was performed in compliance with relevant regulatory standards and was approved by the MSKCC Institutional Animal Care and Use Committee.

### Generation of *Baz1a* conditional mice

The targeting vector was constructed by PCR-amplifying short (∼2 kb) and long (∼5 kb) homologous flanking fragments from bacterial artificial chromosome (BAC) DNA (RP23-235B6) with synthetic *Spe*I-*Hind*III and *Not*I-*Hind*III sites respectively at their ends and cloning into vector PL253 [81] at *Spe*I and *Not*I sites. The BAC *E. coli* strain was electroporated with the pRed/ET recombineering plasmid (Gene Bridges) and PL253 containing the homology arms linearized at *Hind*III to capture a partial genomic *Baz1a* sequence (∼12 kb) containing exons 5–7 into PL253 by homologous recombination upstream of thymidine kinase (TK). Next, short arms of homology were PCR amplified from intronic sequence between *Baz1a* exons 5 and 6 with a synthetic 3′ EcoRI site on the left arm and a synthetic 5′ *Bam*HI site on the right arm and inserted into *Eco*RI and *Bam*HI sites of PL452 [81], which contains a neomycin-resistance cassette (*Neo*) flanked by *FRT* sites with a single *loxP* site downstream. The recombineering strain EL350 [82] was used to insert this construct into the intron upstream of exon 6 of the partial *Baz1a* locus in PL253. A second *lox*P site was inserted into a unique *Avr*II site in the intron downsteam of exon 6.

The targeting construct was linearized at the *Sal*I site, electroporated into albino C57BL/6J, CY2.4 ES cells and *Neo^r^* clones were selected using G418. Individual clones were analyzed by Southern blot using 5′ and 3′ diagnostic probes (Figure S3). Targeted ES cell clones were expanded and injected into C57BL6/J blastocysts. One injected clone (clone #73) successfully contributed to the germ line to generate chimeras heterozygous for the *Baz1a^flox^* allele. *Baz1a^flox^* mice were crossed to an FVB/NJ *Stra8-Cre* transgenic mice to delete exon six in the germ line [41]. Resulting *Baz1a^+/−^* animals were bred to generate homozygous *Baz1a^−/−^* mice on a mixed C57Bl/6J x FVB/NJ background. Mice described in this study were maintained on this mixed background by mating brothers and sisters. To minimize variability from strain background, experimental animals were compared to controls from the same litter or from the same matings involving closely related parents. Adult mice between 2–6 months of age were used in all experiments unless otherwise noted. Genotyping was performed by PCR using primers to detect the *Baz1a* ^flox^ allele: primers P2 (5′-AAACAGGTGGAGAACTTGG) and P3 (5′-CACAGGCATATGCTACCTAGG), which amplify fragments of 245 bp for the wild-type allele and 411 bp for the mutant (Figure 2A). Recombination of *loxP* sites by Cre recombinase was confirmed using primers P1 (5′-TTCCTCGTGCTTTACGGTATCG), P2 and P3, which amplify fragments of 245 bp for the wild-type allele and 331 bp for the mutant allele (Figure 2A). PCR conditions were as follows: 1 minute at 95°C, then 33 cycles of 20 s at 95°C, 30 s at 55°C, and 45 s at 72°C, followed by a final extension for 3 minutes at 72°C.

### RNA expression analysis

To separate spermatids and spermatocytes using FACS, testes from adult mice were processed as described elsewhere [83]. Briefly, cells were liberated from testes by enzymatic treatment with collagenase, trypsin and DNase I and the resulting cell suspension was stained with Hoechst 33342 (Sigma) and sorted using a MoFlo cytometer (Dako) based on red and blue Hoechst fluorescence (reflecting DNA content and chromatin complexity). The purity of enriched populations was determined based on cellular morphology (round spermatids) or immunofluorescence (IF) for SYCP3 (primary spermatocytes) on squash preparations of sorted cells (Table S1; data not shown).

For microarray gene expression profiling, RNA was extracted using TRIzol (Invitrogen) according to the manufacturer's instructions from sorted cells. RNA samples were labeled and hybridized to the MouseWG-6 v2.0 Expression BeadChip microarray (Illumina). All analyses were performed on probe-level data exported by Illumina BeadStudio software. Microarray data were background corrected by the norm-exponential procedure using control probes on the array, quantile-normalized, and analyzed for differential expression by linear model with array weights using the limma package [84] in Bioconductor (http://www.bioconductor.org) and R (http://cran.r-project.org), following recommendations described in the BeadArrayUseCases Bioconductor package [85], [86]. Probe annotations were corrected and poor quality probes removed from analysis as described [87] using Bioconductor package illuminaMousev2.db version 1.14.0. Only probes with detectable expression in at least one sample were included in the differential expression analysis. Purity information was unavailable for one of the *Baz1a^−/−^* spermatocyte samples (mouse 4), and expression data indicated that this sample contained substantial contamination with spermatids (data not shown), so this sample was censored from the tests for differential expression. Unless otherwise indicated, cutoffs of two-fold expression difference and FDR q = 0.05 were applied to define probes as differentially expressed, but overall patterns discussed here remained unchanged if stringency was relaxed to 1.5-fold change and/or q = 0.1 (data not shown). GO term enrichment was analyzed using the WebGestalt server [88]. Data are available under GEO accession number GSE41303.

For quantitative real time PCR (qRT-PCR), RNA was extracted from FACS sorted populations of either spermatocytes or spermatids using TRIzol (Invitrogen) according to the manufacturer's instructions and cDNA was produced using SuperScript III First-Strand Synthesis System (Invitrogen) with oligo-dT as the primer. The amplifications were performed using a LightCycler 480 II (Roche) under the following conditions: 5 minutes at 95°C, then 60 cycles of 10 s at 95°C, 20 s at 55°C, and 30 s at 72°C. Primers used for repetitive element analysis by qRT-PCR were as described [89]. PCR amplification of the cDNA was used to generate gene specific amplicons that were then gel purified. Each amplicon was then serially diluted by a factor of 10 to create a dilution series ranging from 10^−2^ to 10^−6^ to generate a standard curve by qPCR specific for each primer pair against which the relative expression of each unknown was measured.

The pachytene piRNA northern was performed using MIWI-bound RNA isolated from an immunoprecipitation (IP) of testis lysate. Testes were lysed in 1 mL RIPA buffer (50 mM Tris-HCl, pH 7.4; 150 mM NaCl; 1% NP40; 0.25% sodium deoxycholate), and homogenized with a plastic pestle prior to to pelleting cellular debris at 15,000 rpm for 10 minutes. Supernatants were incubated with 10 µL anti-MIWI antibody (Cell Signalling # 2079) overnight at 4°C and bound with 50 µL protein-G agarose. Beads were washed three times for 5 minutes each with 1 mL RIPA buffer before RNA was extracted using TRIzol (Invitrogen) according to the manufacturer's instructions. RNAs were separated on a denaturing 12% polyacrylamide gel at 300 V and transferred to Genescreen Plus by semi-dry transfer in 0.5× TBE for 1 hr at 300 mA. The blot was cross-linked at 1200 µJ and blocked with hybridization buffer (5× SSC, 1 mM EDTA, 2× Denhardt's, 1% SDS, 2% dextran sulfate, 30 µg/mL ssDNA) at room temperature for 10 minutes and then at 42°C for 20 minutes and probed overnight at 42°C with a [γ-^32^P]dATP labeled mixture of oligonucleotides directed against pachytene piRNAs piR-1,2,3 [90]. The blot was washed four times for 30 minutes each with 2XSSC, 0.1% SDS at 42°C followed by exposure to a phosphoimaging screen overnight.

### Histology and cytology

Sperm were isolated from epididymides by mincing in PBS and allowing the sperm to swim or diffuse out. Sperm were counted by hemocytometer and a drop of suspension was added to a slide coated with 4% PFA, air-dried and rinsed with PBS for viewing by bright field microscopy.

For histology, testes or epididymides from adult mice were fixed overnight in 4% paraformaldehyde (PFA) or Bouin's fixative. Ovaries were fixed in 4% PFA for 1 hr. Tissue was embedded in paraffin and 5 µm sections were cut and mounted on slides for either staining with hematoxylin and eosin or periodic acid Schiff or for immunofluorescence or immunohistochemistry using the Ventana Medical Systems Discovery XT automated stainer. Surface spread spermatogenic cells were prepared as described elsewhere [91] as were squashes [92]. Spreads, and squashes were incubated with blocking buffer (1× PBS with 0.2% gelatin from cold-water fish skin, 0.05% TWEEN-20, 0.2% BSA) at room temperature with gentle agitation for 30 min and stained with primary antibodies diluted in blocking buffer. Slides were incubated with primary antibody at 4°C overnight followed by three 5-minute washes in blocking buffer with gentle agitation, incubation with the appropriate AlexaFluor secondary antibody (Invitrogen) diluted 1∶100, washed three times again and mounted with cover slips using Vectashield mounting medium containing 4′,6-diamidino-2-phenylindole (DAPI). Primary antibodies and their dilutions were as follows: anti-BAZ1A (Sigma HPA002730), 1∶100 dilution; anti-SNF2H (Novus H00008467), 1∶100 dilution; anti-HP1γ (Millipore MAB3450), 1∶100 dilution; anti-HP1β (Millipore MAB3448), 1∶100 dilution; anti-H3K9me3 (Abcam ab8898), 1∶1000 dilution; anti-CHRAC17 (Novus NB100-61082) 1∶100 dilution; anti-acetylH4 (Millipore 06-866) 1∶100 dilution; anti-γH2AX (Millipore 05-636) 1∶100 dilution; anti-MLH1 (BD Pharmingen 51-1327GR) 1∶50 dilution; anti-DMC1 (Santa Cruz sc-22768) 1∶100 dilution; anti-SYCP3 (Santa Cruz sc-74569), 1∶50 dilution; anti-MVH (Abcam ab13840).

Terminal deoxynucleotidyl transferase (TdT) dUTP nick end labeling (TUNEL) was perfomed on 4% PFA-fixed testis sections as described [93].

For electron microscopy, testes from adult mice were fixed overnight in 2.5% glutaraldehyde and 2% PFA in 0.075 M sodium cacodylate buffer (pH 7.5). Tissues were then post-fixed in 1% osmium tetroxide, dehydrated, embedded in resin and ultra-thin sections cut and stained with 2% uranyl acetate and Reynolds lead citrate and mounted on copper grids for evaluation using a JEOL1230 transmission electron microscope.

### DR-GFP and MMC experiments

For cell culture, MEFs were derived from 13.5-d embryos, and ear fibroblasts were derived from 2- to 4-mo-old animals. For transfection experiments, mice or embryos were *Pim1^+/Drgfp^*. Embryos were harvested and primary cultures of MEFs were prepared as described in [51] with the following modifications: trunks were minced with a sterile razor blade and dissociated in 1 mL of 0.05% trypsin/EDTA at 37°C for 45 min followed by pipetting up and down several times in 4 mL DME-HG/10% FBS/1% Pen-Strep. Ears were dissociated by agitation in an orbital shaker at medium speed for 4 h in DME-HG with 2 mg/mL collagenase (Roche). After shaking, cells were passed through a 70 µm cell strainer (Falcon) into 50 mL conical tubes and pelleted by centrifugation at 300× g for 5 min. Primary fibroblasts derived from each mouse (2 ears) were plated on a 60 mm plate in DME-HG/10% FBS/1% Pen-Strep. MEFs (3×10^6^) and ear fibroblasts (2–3×10^6^) at passage 3 were electroporated in 0.6 mL Optim-MEM (Gibco) at 350 V and 1000 µF with 30 µg I-SceI expression vector (pCBASce) or empty vector (pCAGGS) or for a positive control 30 µg NZEGFP using a Bio-Rad Gene Pulser II. Flow cytometry was performed 48 h after transfection to analyze GFP expression.

For MMC experiments, 6 mutant and 6 control mice (2- to 4-mo-old) received intraperitoneal injections of 5 mg/kg body weight MMC (Sigma) and were monitored for 21 d.

### SDS-PAGE and western blots

For IP-western and co-IP, a single testis per IP from an adult mouse was homogenized in hypotonic lysis buffer (20 mM HEPES-NaOH, pH 7.5; 5 mM KCl) using a plastic pestle, lysed by freeze/thawing thrice in liquid nitrogen and 37°C water bath for 30 s each and treated with 25 units of benzonase (Novagen) at 4°C for 1 hr. NaCl was increased to 500 mM with 5 M NaCl for 10 min and then reduced to 150 mM by adding dH_2_O. Lysates were incubated with antibody overnight and incubated with protein-G beads (Roche) for 1 hr. Beads were washed three times with PBS and boiled in 2× Laemmli buffer prior to separation on 3–8% Tris-acetate polyacrylamide gels (Invitrogen) at 150 V. Proteins were transferred to PVDF by wet-transfer in Tris-glycine at 100 V for 1 hr at 4°C. Membranes were blocked with 5% non-fat milk in TBS-T (1× TBS with 0.1% TWEEN-20) at room-temperature for 1 hr and incubated with antibodies overnight at 4°C (as listed for histology and cytology but diluted 1∶1000 in block buffer), washed three times for 5 minutes each with TBS-T, and subsequently detected with HRP-congugated secondary antibodies (diluted 1∶10,000 in block) for 1 hr at room-temperature followed by exposure to film.

For multi-tissue western blot, ∼100 mg pieces of a panel of adult mouse organs were flash frozen in liquid nitrogen and homogenized in RIPA buffer by first mincing with scissors and then grinding with a plastic pestle. Lysates were treated with 25 units of benzonase at 4°C for 1 hr and sonicated alternating 30 sec on high setting, 30 sec off for 15 min using a water bath sonicator at 4°C (Diagenode Bioruptor). Protein concentrations were measured using the Bio-Rad protein assay and 8.5 µg of each lysate was added to an equal volume of 2× Laemmli buffer and boiled prior to separation on 3–8% Tris-acetate gels (Invitrogen) and transferred to PVDF by wet-transfer in Tris-glycine as described above. The membrane was stained with Ponceau-S to evaluate protein loading prior to incubation with antibodies.

For cell culture extracts, immortalized ear fibroblasts were generated by mincing the dorsal tip of wild-type and *Baz1a* ^−/−^ mouse ears in DMEM with collagenase D/dispase (4 mg/mL) and incubating at 37°C for 2 hr. Following the incubation, 1.5 mL DMEM with 10% fetal bovine serum and 5× antibiotic/antimycotic (Gemini Bioproducts) was added. Following an overnight incubation at 37°C, tissue was dissociated by pipetting up and down ∼30× with a P1000. Cells were passed through a 40 µm cell strainer, pelleted and resuspended with 3 mL DMEM with 10% fetal bovine serum and 5× antibiotic/antimycotic and plated in a single well of a 6-well dish and incubated at 37°C with 5% CO_2_. After 2 days, the media was changed with the amount of antibiotic/antimycotic reduced to 2×. For simian virus 40 (SV40) transformation, 10 cm dishes containing subconfluent early-passage ear fibroblasts (EFs) were each transfected with 5 µg of the p129-SV40 plasmid (kind gift from Janet Mertz, University of Wisconsin) using FuGENE 6 (Roche) transfection reagent according to the manufacturer's instructions. Immortalized cells were selected for by passaging the cells 10 times. To derive the extracts, the various cell lines were grown to confluency in DMEM +10% fetal calf serum on 10 cm plates, scraped and pelleted by centrifugation and processed like the tissue samples except 25 µg of each extract was used. Additional antibodies: anti-BAZ1B antibody (Sigma # W3516), diluted 1∶1000; anti-actin [ac-15] (Abcam # ab6276), diluted 1∶2500.

For cellular fractionation, testes were dissociated enzymatically by incubating with 4 mg collagenase in 10 mL Gey's balanced salt solution (GBSS) in a thermomixer R (Eppendorf) at 32°C at 450 rpm for 15 min and mechanically by pipetting up and down using a transfer pipette. The resulting single cell suspension was fractionated as described elsewhere [94]. Samples were run on 4–12% Bis-Tris gels (Invitrogen) and transferred to PVDF by wet-transfer in Tris-glycine as described above. The following antibodies were used for western analysis: anti-MacroH2A1.2 (kind gift from John Pehrson, University of Pennsylvania), diluted 1∶1000; anti-Hils1 (kind gift from Martin Matzuk, Baylor College of Medicine), diluted 1∶2000; anti-H2A.Bbd (Millipore 06-1319), diluted 1∶1000; anti-H2AZ (Millipore 07-594), diluted 1∶2000; anti-H3.3 (Abcam ab62642), diluted 1∶1000; anti-Tubulin (Santa Cruz sc-5286), diluted 1∶5000.

### Acid-Urea PAGE analysis of sperm basic nuclear proteins

Testes were dissociated and nuclei were isolated as described above for the cellular fractionation. Nuclei were then sonicated alternating 30 sec on high setting, 30 sec off for 15 min using a water bath sonicator at 4°C (Diagenode Bioruptor) and centrifuged at 6000× g for 5 minutes. The supernatant (sonication-sensitive fraction) and pellet (sonication-resistant nuclei fraction) were collected separately. Proteins soluble in 500 mM hydrochloric acid were isolated from each fraction and precipitated with 4% and then 20% trichloracetic acid. Precipitates were washed with 500 µL acetone and dried under vacuum, then boiled in 100 µL loading buffer (5.5 M urea, 5% acetic acid, 20% β mercaptoethanol) and separated on a 2.5 M urea, 0.9% acetic acid, 15% polyacrylamide gel (which was pre-run overnight in reverse polarity at 150 V) in reverse polarity at 150 V in 5% acetic acid. The gel was stained with Coomassie and destained with 50% methanol, 10% acetic acid.

### B cell purification and *in vitro* stimulation of CSR

Splenic B cells were purified using negative selection with anti-CD43 magnetic beads (Miltenyi Biotec) and stimulated with LPS to induce CSR to IgG3 or anti-CD40+IL4 at a density of 10^6^ cells/mL in RPMI 1640 supplemented with 15% fetal calf serum, 2 mM L-glutamine, 100 IU/mL penicillin, 100 µg/mL streptomycin, 60 µM beta-mercaptoethanol. Activated B cells were re-fed at 48 hours with fresh stimulation medium and analyzed by flow cytometry at 96 hours post-stimulation on a FACSAria flow cytometer (BD Biosciences). Antibodies used for flow cytometry were from BD Biosciences: anti-IgG1-APC (#550874) and anti-IgG3-FITC (#553403). Reagent concentrations were 20 µg/mL LPS (Sigma L4130), 1 µg/mL anti-CD40 (eBiosciences 16-0402-86), and 12.5 µg/mL IL4 (R&D Systems 404-ML-050).

### FACS analysis of CD4+ and CD8+ T cell populations

Single-cell suspensions of thymocytes and splenocytes were generated by dissecting the organs and grinding each between the frosted-portion of glass slides into 5 mL Bruff's medium with 5% fetal bovine serum. Cells were pelleted at 1500 rpm for 5 minutes. To the splenocytes, 1 mL red blood cell lysis buffer (Sigma) was added and incubated at room-temperature for 10 minutes. Splenocytes were pelleted and resuspended in 5 mL Bruff's medium. Both cell suspensions were counted by hemocytometer and plated at 10^6^ cells/well in a 96-well plate. Each well was incubated with normal mouse serum and 75 µg Fc receptor-blocking antibody (Miltenyi Biotec) at 4°C for 20 minutes before being stained at 4°C for 30 minutes with 50 µL/well of monoclonal antibody conjugates from BD Biosciences: anti-CD4-Pacific Blue (#558107) and anti-CD8-FITC (#561968). Stained suspensions were analyzed using a FACSAria flow cytometer (BD Biosciences).

### ICSI (Intracytoplasmic sperm injection) and ROSI (round spermatid injection)

Round spermatids were isolated based on their morphology in bright field microscopy, from a suspension of *Baz1a* ^−/−^ testis cells generated by enzymatic treatment of a pair of decapsulated testes with collagenase, trypsin and DNase I. Sperm were isolated from epididymides by mincing in PBS and allowing the sperm to diffuse out. *Baz1a^−/−^* round spermatids or sperm heads were injected into oocytes retrieved from BDF1 females 13 hours after injection with human chorionic gonadotropin. All manipulations were performed on the heated stage of a Nikon TE2000 microscope equipped with a Narishige micro-manipulator. Oocytes were monitored for 6 hours post injection and scored for signs of activation, namely, polar body extrusion and the appearance of interphase nuclei. As neither round spermatids nor sperm heads activated the oocytes, artificial activation of injected oocytes was performed by incubation in 10 mM strontium chloride in calcium-free MCZB. Embryos were cultured to the blastocyst stage in Global total in a MINC incubator containing 5% O_2_, 6% CO_2_, 89% N_2_.

## Supporting Information

Figure S1 Cross-species comparison of ISWI containing complexes and ACF orthologs and paralogs. (A) Subunit compositions of known ISWI-containing chromatin remodeling complexes from *Drosophila* and mammals. Like colors represent orthologous proteins. Patterned after ref [95] (B) Comparison of the domain architecture of Acf1 from *Drosophila* and the mouse BAZ/WAL family of proteins. Numbers at the right indicate the amino acid length of each peptide. Not to scale. (C) Amino-acid sequences of BAZ1A (red) and its seven paralogs from primate (human and chimpanzee), rodent (mouse and rat), bird (chicken), laurasiatheria (dog), fish (fugu) and amphibian (frog) were aligned with Acf1 from fruitfly and Itc1 (blue) from the budding yeast *Saccharomyces cerevisiae* by the Clustal V method and the tree constructed using MegAlign software. Dotted line = negative branch length.
(PDF)

Figure S2 Expression and localization of BAZ1A during spermatogenesis and oogenesis. (A) Staging of the seminiferous epithelium is based on the semi-synchronous waves of spermatid differentiation along the length of seminiferous tubule. A single seminiferous tubule from the tesis is enlarged with an example of a fluorescent-stained cross-section at the end. Arrow indicates the direction of the semi-synchronous wave of spermatogenesis with roman numerals indicating the 12 epithelial stages. (For further explanation of staging, please see [96]). (B) Immunofluorescence on mouse testis sections. (i–iv) Sections were co-stained with anti-BAZ1A and HP1β antibodies. Tubule stage is indicated in uppercase roman numerals. (i′–iv′) Same images as panels above with increased exposure in the BAZ1A (red) channel alone. Bar = 20 µm. Insets show magnification of the indicated cells. DAPI was included in the insets in the bottom panel to show nuclear shape. Lowercase letters to the left of each inset correspond to the letters in Figure 1D. (C) Immunofluorescence comparing BAZ1A expression in testis and ovary sections. Sections from adult testis and embryonic ovary (∼18.5 days post-conception) were stained in parallel with anti-SYCP3 and BAZ1A antibodies. Fluorescent signals in the BAZ1A channel were captured with equal exposures and displayed with the same contrast settings to provide semi-quantitative comparison. Note that BAZ1A staining in pachytene or diplotene oocytes (O) is relatively weak, especially compared to the overexposed signal in pachytene spermatocytes (P). Weak, variable BAZ1A signal was also observed in diplotene or dictyate oocytes from 2 dpp ovaries (data not shown). Moreover, the oocyte signal is specific for BAZ1A, as no such staining was detected in oocytes from *Baz1a^−/−^* animals. RS, round spermatids; pL, pre-leptotene spermatocytes; bar = 10 µm. (D) BAZ1A immunofluorescence on spread spermatocyte nuclei at different stages of prophase I of meiosis as indicated by accumulation of the axial element component SYCP3. Arrows, sex bodies; bar = 5 µm.
(PDF)

Figure S3 Confirmation of *Baz1a* disruption. (A) Genomic locus and targeting strategy reproduced from Figure 2A, with restriction map and Southern blot probes (red boxes overlaid). Not to scale. (B) Southern blots confirming successful gene targeting in six CY2.4 ES cell clones, designated by the numbers above each lane. Clone 73 generated the mice used in this study. The expected digest fragment sizes are indicated at the right in kb. M, marker; CY2.4, untargeted ES cell DNA serves as a negative control. Asterisk indicates a cross-reacting band. (C) CHRAC17 and SNF2H were immunoprecipitated (IP) from wild-type and mutant whole testis lysates and immunoblotted for co-IP with BAZ1A, SNF2H and CHRAC17. Mock IP, no antibody. CHRAC17 was not detectable in anti-SNF2H immunoprecipitates, but we note that SNF2H forms multiple complexes that do not include CHRAC17 (including ACF) (Figure S1A). Moreover, BAZ1A was less efficiently co-precipitated with anti-SNF2H than with anti-CHRAC17, and anti-SNF2H and anti-CHRAC17 both precipitated similar amounts of SNF2H. We thus consider it likely that the anti-SNF2H immunoprecipitation is overall less efficient and contains a mixture of different ISWI complexes, many of which lack BAZ1A, CHRAC17 or both, rendering CHRAC17 below the limit of detection.
(PDF)

Figure S4 Heterochromatin formation appears normal in the absence of *Baz1a*. Immunofluorescence on squash preparations of pachytene/diplotene spermatocyte nuclei (A–C) or round spermatids (D–F) from wild type and mutant with antibodies against the heterochromatin markers H3K9me3 (panels A, D), HP1β (panels B, E) and HP1γ (panels C, F). Spermatocytes are also stained for SYCP3. The DAPI-bright regions are the pericentric heterochromatin, which forms numerous discrete clumps in spermatocytes but coalesces in round spermatids into a single condensed structure, the chromocenter. Bar = 10 µm.
(PDF)

Figure S5 Chromosome-specific expression patterns. (A, B) MA plots for probes mapping to the X or Y chromosomes, comparing average expression level to fold change in expression for control spermatids vs spermatocytes (A) or for Baz1a^−/−^ vs. control spermatids (B). The substantial up regulation of many X and Y probes that occurs in the spermatocyte-to-spermatid transition in control animals (A) occurs normally in the absence of BAZ1A (B). Only probes that scored as detectably expressed in at least one sample analyzed in this study are plotted. Dotted line, no change in expression; dashed lines, two-fold change in expression. (C) Chromosomal distribution of differentially expressed probes. For each probe set, the number of probes mapping to a given chromosome was compared to the number expected by chance given how many probes from that chromosome were present on the microarray. Asterisks, statistically significant under- or over-representation (Fisher's exact test, p≤0.05 after correction for multiple testing). No autosomes showed significant enrichment or depletion of probes that are differentially expressed in the normal spermatocyte-to-spermatid transition (“developmentally regulated”), but sex chromosomes were significantly overrepresented. In contrast, sex chromosome probes were greatly underrepresented among those mis-regulated in *Baz1a^−/−^* spermatids (cf. panel B). Probes from chromosomes 1 and 16 were also overrepresented in the mis-regulated class, but the biological significance of this pattern is not known.
(PDF)

Figure S6 Normal expression of pachytene piRNAs and repetitive elements in *Baz1a*-deficient spermatids. (A) Northern blot of MIWI-bound RNA following immunoprecipitation and detection with a mixed probe of the pachytene-piRNAs piR-1, 2 and 3. WCE, whole cell extract; IP, immunoprecipitate; M, marker; mock IP, no antibody. (B) Quantitative real-time PCR analysis of the indicated repetitive elements from wild-type and mutant spermatids (mean ± s.d).
(PDF)

Table S1 Purity of sorted testis cell populations.
(PDF)

Table S2 Lists of differentially expressed genes from microarray analysis (Excel file).
(XLSX)
