# Supplementary figures and images for: Mouse BAZ1A (ACF1) Is Dispensable for Double-Strand Break Repair but Is Essential for Averting Improper Gene Expression during Spermatogenesis
Source: PLoS Genet. 2013 Nov 7;9(11):e1003945. doi: 10.1371/journal.pgen.1003945 (PMC3820798; doi:10.1371/journal.pgen.1003945)

FIGURE S1.

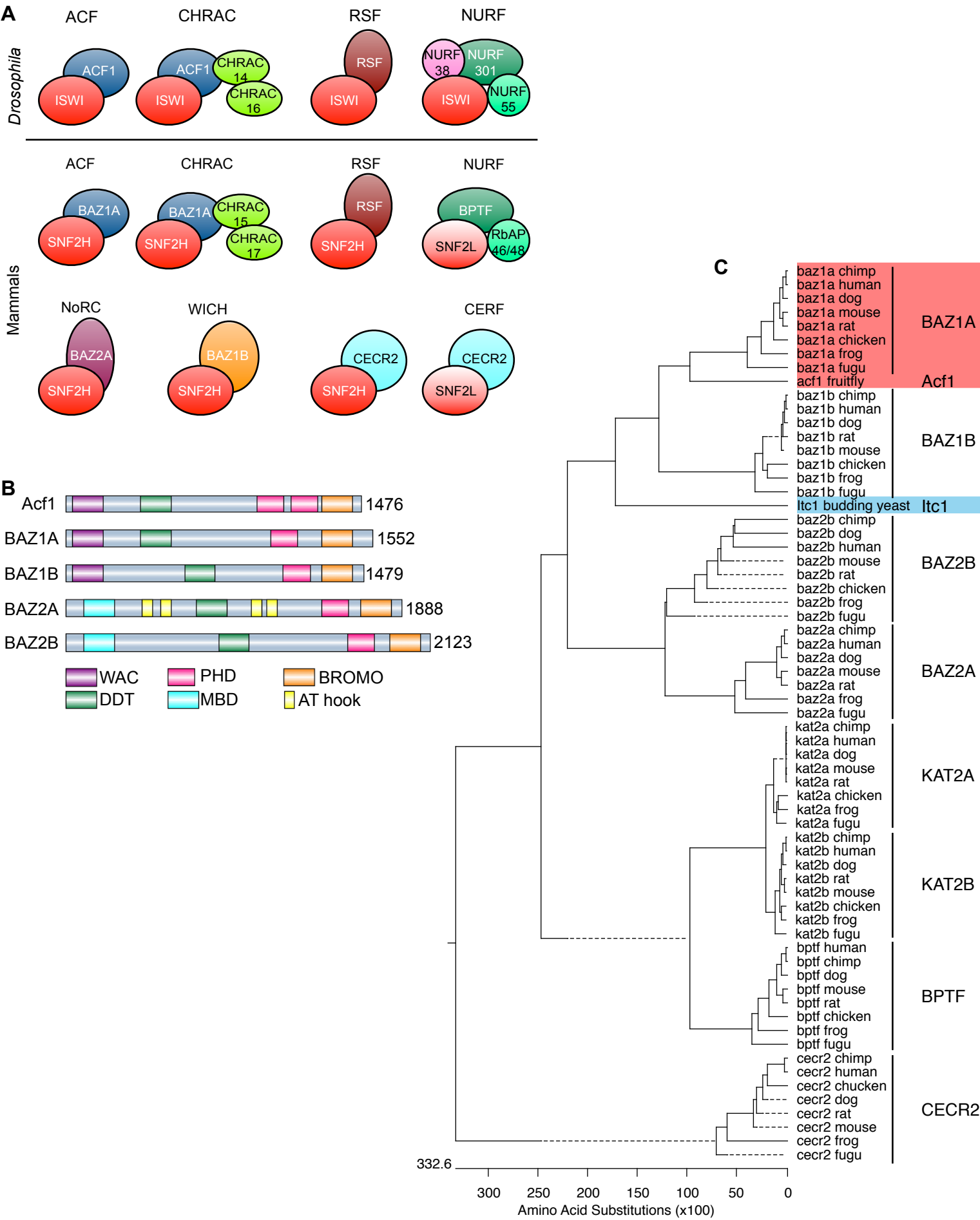

Supplement: Figure S1 — Cross-species comparison of ISWI containing complexes and ACF orthologs and paralogs. (A) Subunit compositions of known ISWI-containing chromatin remodeling complexes from Drosophila and mammals. Like colors represent orthologous proteins. Patterned after ref [95] (B) Comparison of the domain architecture of Acf1 from Drosophila and the mouse BAZ/WAL family of proteins. Numbers at the right indicate the amino acid length of each peptide. Not to scale. (C) Amino-acid sequences of BAZ1A (red) and its seven paralogs from primate (human and chimpanzee), rodent (mouse and rat), bird (chicken), laurasiatheria (dog), fish (fugu) and amphibian (frog) were aligned with Acf1 from fruitfly and Itc1 (blue) from the budding yeast Saccharomyces cerevisiae by the Clustal V method and the tree constructed using MegAlign software. Dotted line = negative branch length. (PDF) [file pgen.1003945.s001.pdf]

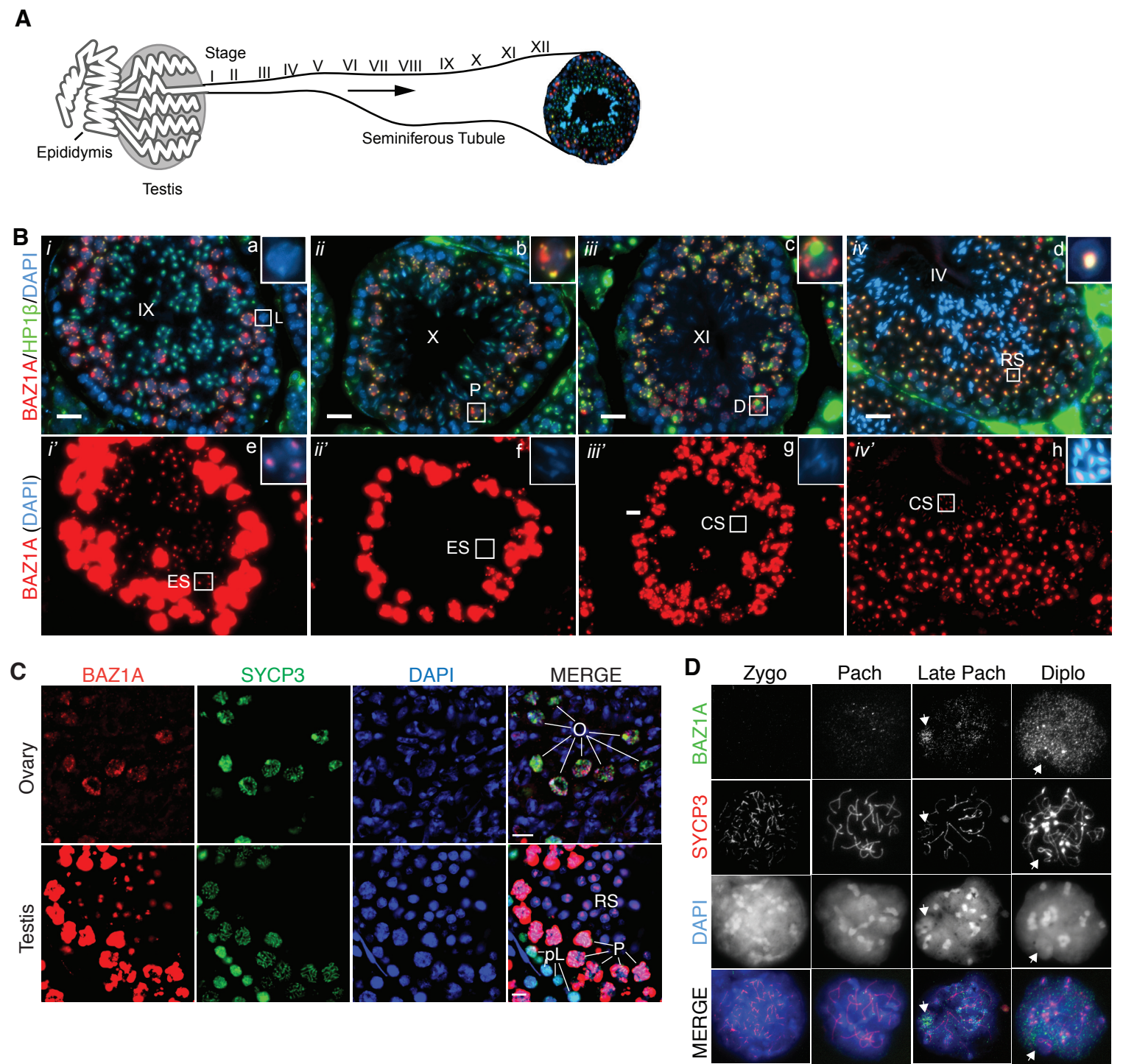

Supplement: Figure S2 — Expression and localization of BAZ1A during spermatogenesis and oogenesis. (A) Staging of the seminiferous epithelium is based on the semi-synchronous waves of spermatid differentiation along the length of seminiferous tubule. A single seminiferous tubule from the tesis is enlarged with an example of a fluorescent-stained cross-section at the end. Arrow indicates the direction of the semi-synchronous wave of spermatogenesis with roman numerals indicating the 12 epithelial stages. (For further explanation of staging, please see [96]). (B) Immunofluorescence on mouse testis sections. (i–iv) Sections were co-stained with anti-BAZ1A and HP1β antibodies. Tubule stage is indicated in uppercase roman numerals. (i′–iv′) Same images as panels above with increased exposure in the BAZ1A (red) channel alone. Bar = 20 µm. Insets show magnification of the indicated cells. DAPI was included in the insets in the bottom panel to show nuclear shape. Lowercase letters to the left of each inset correspond to the letters in Figure 1D. (C) Immunofluorescence comparing BAZ1A expression in testis and ovary sections. Sections from adult testis and embryonic ovary (∼18.5 days post-conception) were stained in parallel with anti-SYCP3 and BAZ1A antibodies. Fluorescent signals in the BAZ1A channel were captured with equal exposures and displayed with the same contrast settings to provide semi-quantitative comparison. Note that BAZ1A staining in pachytene or diplotene oocytes (O) is relatively weak, especially compared to the overexposed signal in pachytene spermatocytes (P). Weak, variable BAZ1A signal was also observed in diplotene or dictyate oocytes from 2 dpp ovaries (data not shown). Moreover, the oocyte signal is specific for BAZ1A, as no such staining was detected in oocytes from Baz1a−/− animals. RS, round spermatids; pL, pre-leptotene spermatocytes; bar = 10 µm. (D) BAZ1A immunofluorescence on spread spermatocyte nuclei at different stages of prophase I of meiosis as indicated by accum [file pgen.1003945.s002.pdf]

FIGURE S3.

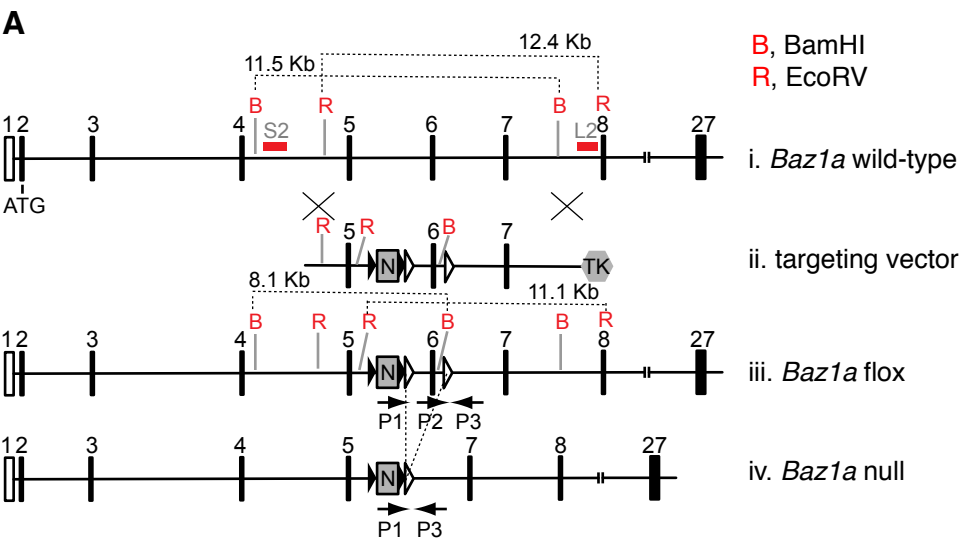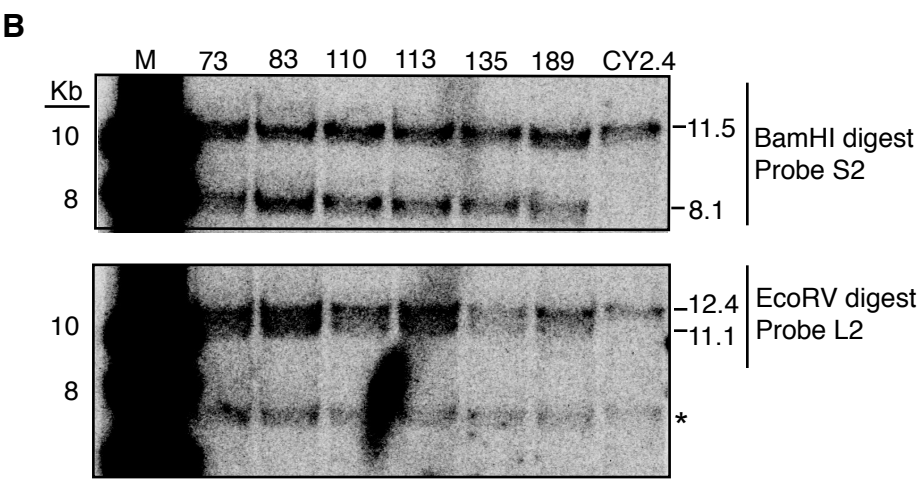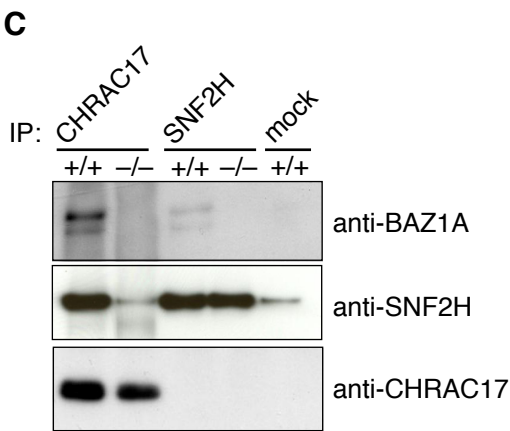

Supplement: Figure S3 — Confirmation of Baz1a disruption. (A) Genomic locus and targeting strategy reproduced from Figure 2A, with restriction map and Southern blot probes (red boxes overlaid). Not to scale. (B) Southern blots confirming successful gene targeting in six CY2.4 ES cell clones, designated by the numbers above each lane. Clone 73 generated the mice used in this study. The expected digest fragment sizes are indicated at the right in kb. M, marker; CY2.4, untargeted ES cell DNA serves as a negative control. Asterisk indicates a cross-reacting band. (C) CHRAC17 and SNF2H were immunoprecipitated (IP) from wild-type and mutant whole testis lysates and immunoblotted for co-IP with BAZ1A, SNF2H and CHRAC17. Mock IP, no antibody. CHRAC17 was not detectable in anti-SNF2H immunoprecipitates, but we note that SNF2H forms multiple complexes that do not include CHRAC17 (including ACF) (Figure S1A). Moreover, BAZ1A was less efficiently co-precipitated with anti-SNF2H than with anti-CHRAC17, and anti-SNF2H and anti-CHRAC17 both precipitated similar amounts of SNF2H. We thus consider it likely that the anti-SNF2H immunoprecipitation is overall less efficient and contains a mixture of different ISWI complexes, many of which lack BAZ1A, CHRAC17 or both, rendering CHRAC17 below the limit of detection. (PDF) [file pgen.1003945.s003.pdf]

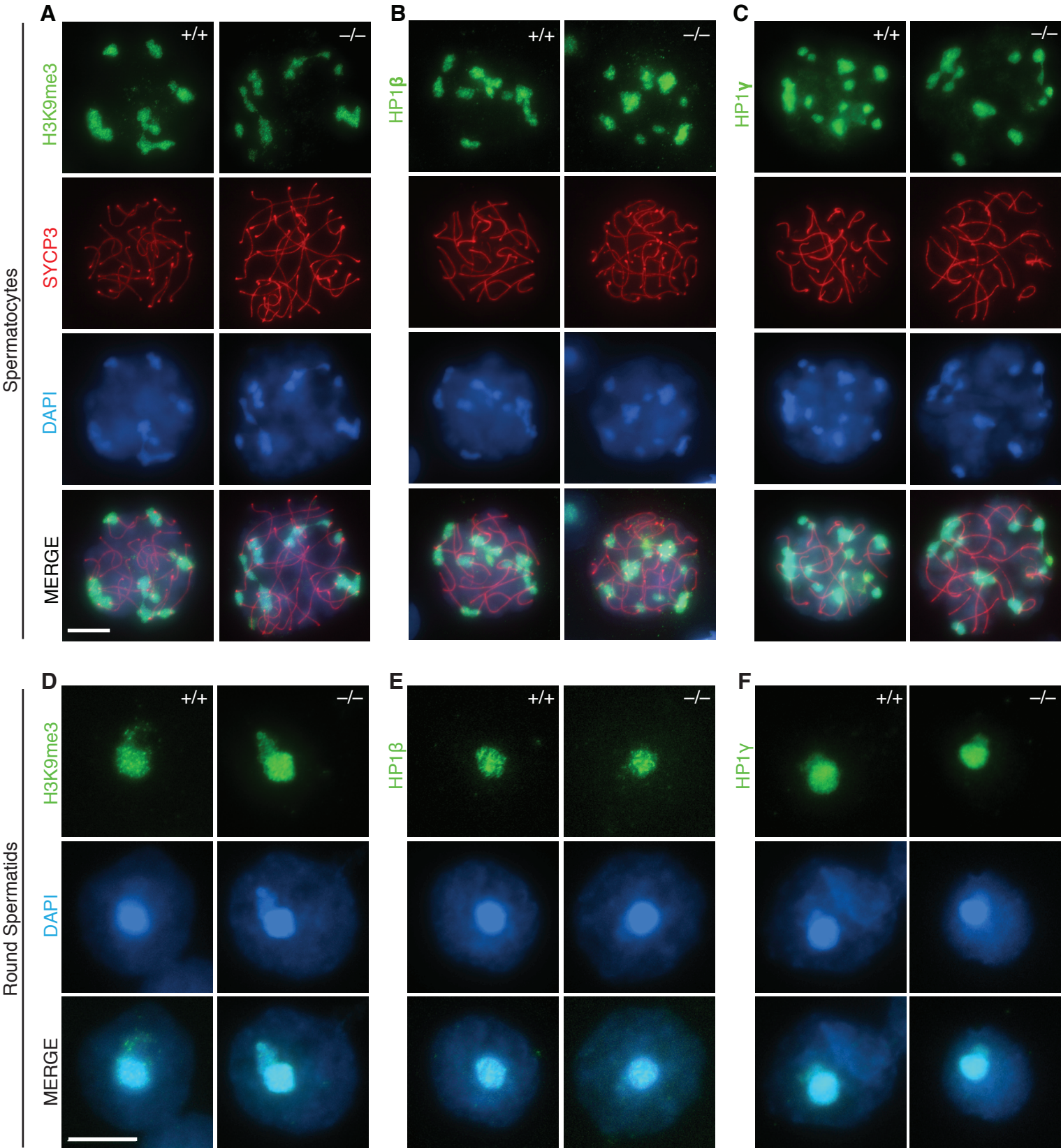

Supplement: Figure S4 — Heterochromatin formation appears normal in the absence of Baz1a. Immunofluorescence on squash preparations of pachytene/diplotene spermatocyte nuclei (A–C) or round spermatids (D–F) from wild type and mutant with antibodies against the heterochromatin markers H3K9me3 (panels A, D), HP1β (panels B, E) and HP1γ (panels C, F). Spermatocytes are also stained for SYCP3. The DAPI-bright regions are the pericentric heterochromatin, which forms numerous discrete clumps in spermatocytes but coalesces in round spermatids into a single condensed structure, the chromocenter. Bar = 10 µm. (PDF) [file pgen.1003945.s004.pdf]

FIGURE S5.

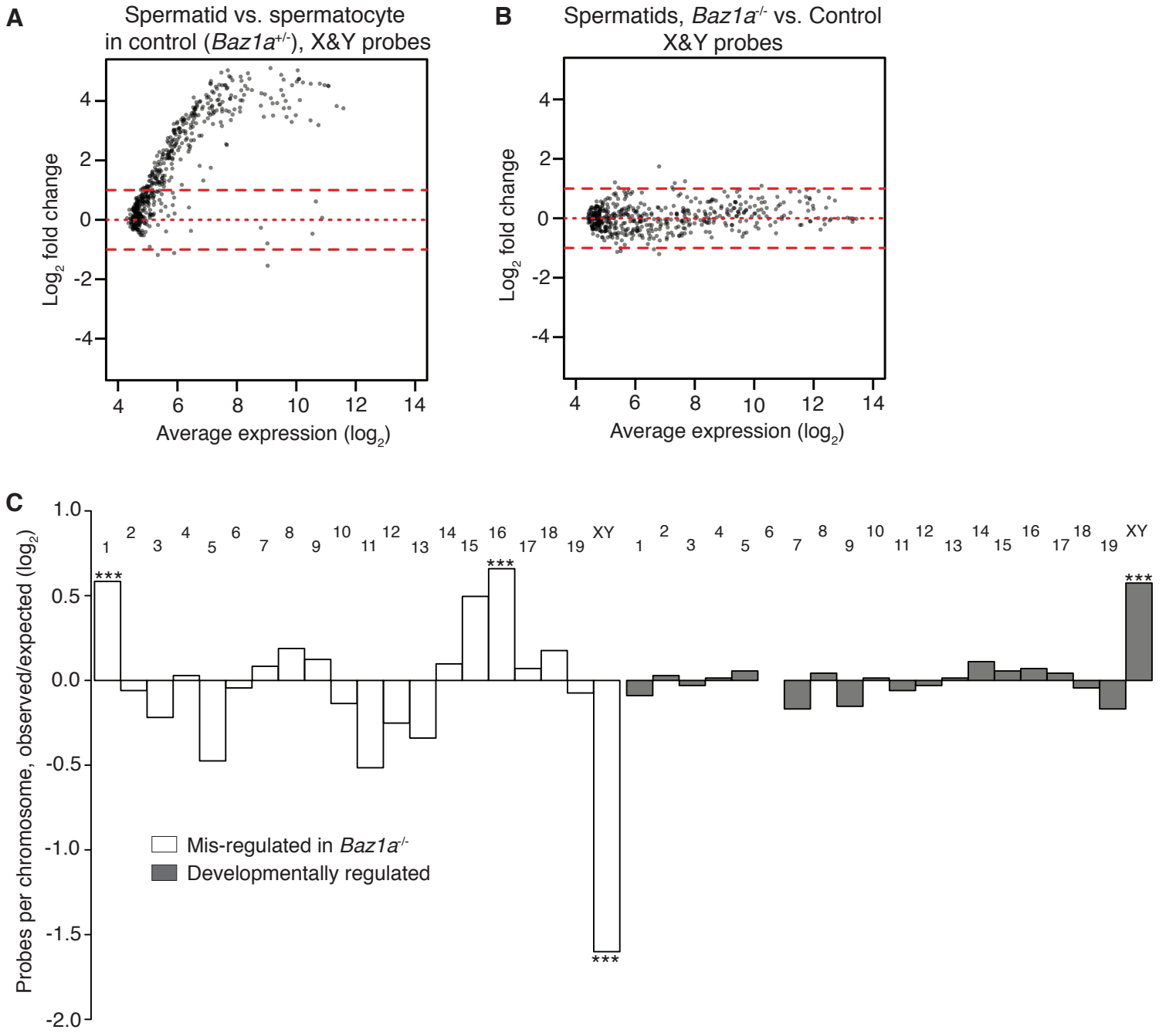

Supplement: Figure S5 — Chromosome-specific expression patterns. (A, B) MA plots for probes mapping to the X or Y chromosomes, comparing average expression level to fold change in expression for control spermatids vs spermatocytes (A) or for Baz1a−/− vs. control spermatids (B). The substantial up regulation of many X and Y probes that occurs in the spermatocyte-to-spermatid transition in control animals (A) occurs normally in the absence of BAZ1A (B). Only probes that scored as detectably expressed in at least one sample analyzed in this study are plotted. Dotted line, no change in expression; dashed lines, two-fold change in expression. (C) Chromosomal distribution of differentially expressed probes. For each probe set, the number of probes mapping to a given chromosome was compared to the number expected by chance given how many probes from that chromosome were present on the microarray. Asterisks, statistically significant under- or over-representation (Fisher's exact test, p≤0.05 after correction for multiple testing). No autosomes showed significant enrichment or depletion of probes that are differentially expressed in the normal spermatocyte-to-spermatid transition (“developmentally regulated”), but sex chromosomes were significantly overrepresented. In contrast, sex chromosome probes were greatly underrepresented among those mis-regulated in Baz1a−/− spermatids (cf. panel B). Probes from chromosomes 1 and 16 were also overrepresented in the mis-regulated class, but the biological significance of this pattern is not known. (PDF) [file pgen.1003945.s005.pdf]

FIGURE S6.

Dowdle, JA

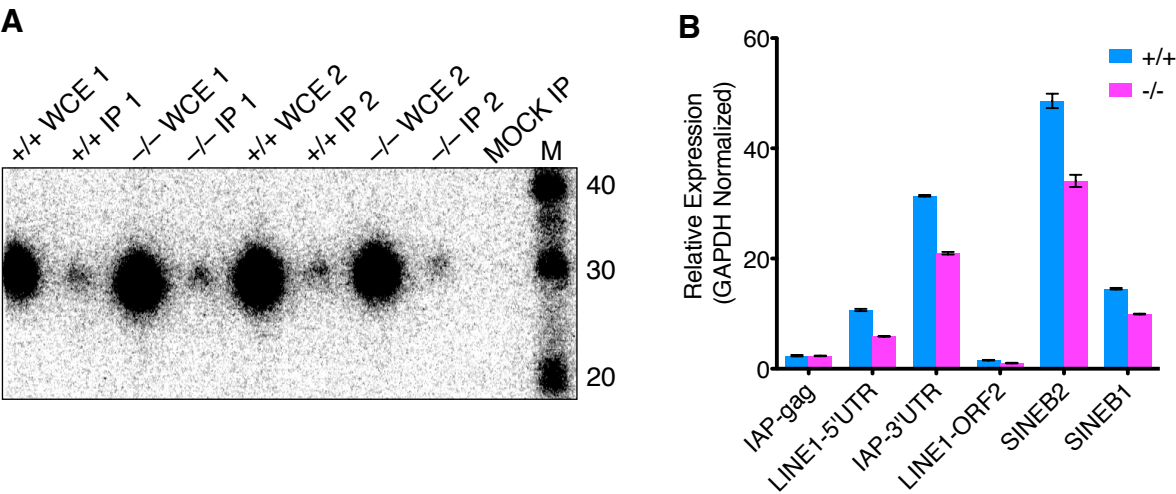

Supplement: Figure S6 — Normal expression of pachytene piRNAs and repetitive elements in Baz1a-deficient spermatids. (A) Northern blot of MIWI-bound RNA following immunoprecipitation and detection with a mixed probe of the pachytene-piRNAs piR-1, 2 and 3. WCE, whole cell extract; IP, immunoprecipitate; M, marker; mock IP, no antibody. (B) Quantitative real-time PCR analysis of the indicated repetitive elements from wild-type and mutant spermatids (mean ± s.d). (PDF) [file pgen.1003945.s006.pdf]
