# Supplementary material for: Mouse BAZ1A (ACF1) Is Dispensable for Double-Strand Break Repair but Is Essential for Averting Improper Gene Expression during Spermatogenesis
Source: PLoS Genet. 2013 Nov 7;9(11):e1003945. doi: 10.1371/journal.pgen.1003945 (PMC3820798; doi:10.1371/journal.pgen.1003945)
Supplement: Table S1 — Purity of sorted testis cell populations. (PDF) [file pgen.1003945.s007.pdf]

Table S1. Purity of sorted testis cell populations.

	Baz1a$^{+/-}$			*Baz1a*$^{-/-}$	
**Mouse**	**P/D Spc.**	**Spermatid**	**Mouse**	**P/D Spc.**	**Spermatid**
1	75%	98%	4	NA	100%
2	91%	100%	5	81%	95%
3	99%	100%	6	100%	100%

Squash preparations of each sorted sample were stained with anti-SYCP3 and DAPI and 100 cells of each were classified based on SYCP3 positivity or spermatid characteristics (i.e. chromocenter). The mouse numbers above correspond to the mouse IDs on the expression data in the GEO repository as follows: 1 = 674, 2 = 710, 3 = 713, 4 = 683, 5 = 709 and 6 = 707. PD Spc., pachytene/diplotene spermatocytes; NA, not applicable because not enough cells were present to accurately quantify.
